# Absence of Apolipoprotein E is associated with exacerbation of prion pathology and promotes microglial neurodegenerative phenotype

**DOI:** 10.1186/s40478-021-01261-z

**Published:** 2021-09-26

**Authors:** Joanna E. Pankiewicz, Anita M. Lizińczyk, Leor A. Franco, Jenny R. Diaz, Mitchell Martá-Ariza, Martin J. Sadowski

**Affiliations:** 1 Department of Neurology, New York University Grossman School of Medicine, 550 First Avenue, Science Building, Room 10-07, New York, NY 10016 USA; 2 Department of Psychiatry , New York University Grossman School of Medicine, New York, NY 10016 USA; 3 Department of Biochemistry and Molecular Pharmacology , New York University Grossman School of Medicine, New York, NY 10016 USA

**Keywords:** Alzheimer’s disease, Apolipoprotein E, Astrocytes, Microglia, Neurodegeneration, Neuroinflammation, Prion diseases, Prion protein

## Abstract

Prion diseases or prionoses are a group of rapidly progressing and invariably fatal neurodegenerative diseases. The pathogenesis of prionoses is associated with self-replication and connectomal spread of PrP^Sc^, a disease specific conformer of the prion protein. Microglia undergo activation early in the course of prion pathogenesis and exert opposing roles in PrP^Sc^ mediated neurodegeneration. While clearance of PrP^Sc^ and apoptotic neurons have disease-limiting effect, microglia-driven neuroinflammation bears deleterious consequences to neuronal networks. Apolipoprotein (apo) E is a lipid transporting protein with pleiotropic functions, which include controlling of the phagocytic and inflammatory characteristics of activated microglia in neurodegenerative diseases. Despite the significance of microglia in prion pathogenesis, the role of apoE in prionoses has not been established. We showed here that infection of wild type mice with 22L mouse adapted scrapie strain is associated with significant increase in the total brain apoE protein and mRNA levels and also with a conspicuous cell-type shift in the apoE expression. There is reduced expression of apoE in activated astrocytes and marked upregulation of apoE expression by activated microglia. We also showed apoE ablation exaggerates PrP^Sc^ mediated neurodegeneration. *Apoe*^−/−^ mice have shorter disease incubation period, increased load of spongiform lesion, pronounced neuronal loss, and exaggerated astro and microgliosis. Astrocytes of *Apoe*^−/−^ mice display salient upregulation of transcriptomic markers defining A1 neurotoxic astrocytes while microglia show upregulation of transcriptomic markers characteristic for microglial neurodegenerative phenotype. There is impaired clearance of PrP^Sc^ and dying neurons by microglia in *Apoe*^−/−^ mice along with increased level of proinflammatory cytokines. Our work indicates that apoE absence renders clearance of PrP^Sc^ and dying neurons by microglia inefficient, while the excess of neuronal debris promotes microglial neurodegenerative phenotype aggravating the vicious cycle of neuronal death and neuroinflammation.

**Supplementary Information:**

The online version contains supplementary material available at 10.1186/s40478-021-01261-z.

## Introduction

Prion disease (or prionoses) are a group of fatal neurodegenerative diseases affecting humans and certain mammalian species. Human prionoses include Creutzfeldt-Jakob disease (CJD), Gerstmann Sträussler Scheinker syndrome, fatal familial insomnia, variably protease-sensitive prionopathy and kuru [33, 52, 63]. Despite relatively low incidence (1/10^6^/year for sporadic CJD), universally fatal outcome, aggressive clinical course (e.g. median survival of 4–5 mos. in sporadic CJD), and absence of treatment make prionoses a devastating condition. Notable examples of animal prionoses (reviewed in [1]) include scrapie in sheep and goats, bovine spongiform encephalopathy, chronic wasting disease in cervine species, and mouse adapted scrapie strains, which are established laboratory models for prionoses [6, 14, 56, 59]. Like Alzheimer’s disease (AD), prionoses belong to neurodegenerative conformational disorders, where the pathogenesis is driven by the accumulation of β-sheet misfolded proteins, which triggers activation of astrocytes and microglia. PrP^Sc^ (Sc-scrapie) is a toxic, proteolysis-resistant, and oligomerization-prone conformer of the cellular prion protein (PrP^C^) typical to all prionoses. PrP^Sc^ replicates by binding to PrP^C^ and altering PrP^C^’s α-helix-rich conformation into the likeness of its own, which is β-sheet-dominant [2, 62]. In infected neurons, PrP^Sc^ accumulates on the cell surface and within the endosomal system [37, 42, 59]. PrP^Sc^ also can be secreted into the neuropil, where it forms diffuse, immunodetectable deposits [14, 22]. Other neuropathological hallmarks of prionoses include spongiform changes reflecting loss of structural integrity of axons, vacuolar changes in neuronal perikarya, and loss of neuronal bodies and synapses [22, 63]. Microglia activation is an early event in prionoses predating occurrence of spongiform lesions. In prionoses, microglia display prominent inflammatory phenotype and secrete numerous cytokines and chemokines, which exert toxic effect on neurons either directly or indirectly by recruiting A1 neurotoxic astrocytes [3, 10]. Conversely, clearance of PrP^Sc^ by microglia, has been proposed as a disease-limiting mechanism though details of engagement, engulfment, and disposal of PrP^Sc^ by microglia remain unknown [12, 29, 30]. The net contribution of microglia proinflammatory vs. phagocytic effect to prion pathogenesis remains actively debated [3, 12].

Apolipoprotein (apo) E is a 34 kDa protein, which is primarily expressed by astrocytes and secreted as high-density lipoprotein (HDL) like particles [18, 25, 40]. ApoE regulates brain lipid metabolism, synaptic plasticity, and synaptic receptor expression [47]. This happens through the interaction of apoE/HDL particles with various neuronal apoE receptors including the low-density lipoprotein receptor (LDLR) [15, 32, 44]. ApoE is nominally expressed by homeostatic or M0 microglia, but *Apoe* transcript becomes steeply upregulated in reactive microglia [36, 43, 45, 69]. High level of *Apoe* expression is a cardinal feature of the transcriptomic profile of disease associated microglia (DAM) [24, 43] and microglial neurodegenerative phenotype (MGnD) [45], which have been isolated from brains of AD transgenic (Tg) mouse models. DAM have been originally identified in *APP* Tg mice serving as a model of Aβ deposition, which represent the initial culprit of AD pathogenesis. Functional significance of DAM is viewed as protective and facilitating clearance of Aβ deposits, despite expression of several inflammatory cytokines [23, 24]. In contrast, MGnD is considered neurodegeneration-promoting due to salient upregulation of proinflammatory signaling [45]. This phenotype has been linked to more advanced stages of AD pathology associated with prominent neurodegeneration and intraneuronal accumulation of hyperphosphorylated tau and it also has been described in mouse models of experimental autoimmune encephalitis, and amyotrophic lateral sclerosis [45]. ApoE controls phagocytic and inflammatory characteristics of microglia in neurodegenerative diseases. Lipidated, astrocyte expressed apoE has been proposed to act as an opsonin facilitating phagocytic function of activated microglia and clearance of cellular debris and misfolded proteins [61, 69, 80, 82]. Conversely, microglia expressed apoE has been suggested to facilitate MGnD maintenance and its pro-inflammatory characteristics [9, 45]. ApoE is critically involved in the pathogenesis of AD where *APOE* genotype both controls AD risk [20, 81] and symptomatic progression [19, 21]. There is an eminent effect of the *APOE ε4* allele on Aβ deposition [16, 39], which determines AD risk. The *ε4* allele also promotes, inflammatory microglia activation, which contributes importantly to the degeneration of tau-bearing neurons downstream to Aβ accumulation [70, 71, 79]. Despite close similarities between AD and prionoses, the role of apoE in the pathomechanism of prion diseases remains unexplored apart from a single study conducted 25 years ago, which primary quantitative readout was the survival of *Apoe*^*−/−*^ mice intracerebrally inoculated with the Rocky Mountain Laboratory (RML) scrapie strain [74]. Regulatory effect of apoE in the activation characteristics of microglia, which play salient role in prion pathogenesis has not been explored. In this study we examined changes in the apoE expression in a murine model of prion disease and the effects of apoE absence on prion pathomechanisms. The overall effect of apoE in prionoses is disease-limiting as apoE facilitates microglia dependent clearance of PrP^Sc^ and degenerating neurons and attenuates neuroinflammation.

## Materials and methods

### Materials and reagents

All chemicals and reagents were purchased from Sigma-Aldrich (St. Louis, MO) unless specifically stated. Sources of antibodies used for immunohistochemistry and Western-blotting along with their working dilution are listed in Tables 1, 2, 3. Polymerase chain reaction primers were synthesized to order by Sigma-Aldrich (Table 4).

**Table 1:** List of primary antibodies used for immunohistochemistry

Antigen	Abbreviation	Host	Dilution	Source	Catalog # (Clone)
Cluster of differentiation 68	CD68	Rat	1:250	Abcam Inc., Cambridge, MA	Ab53444 (FA-11)
Cluster of differentiation 230	CD230	Mouse	1:200	BioLegend, San Diego, CA	808,001 (6D11)
Complement component 3	C3	Rat	1:200	Hycult Biotech, Uden, Netherlands	HM1045 (11H9)
Glial fibrillary acidic protein	GFAP	Rabbit	1:2,000	Dako/Agilent Technologies, Santa Clara, CA	Z0334
Ionized calcium adaptor protein 1	Iba1	Rabbit	1:1,000	Wako Chemicals Inc., Richmond, VA	019–19,741
Murine Apolipoprotein E	apoE	Mouse	1:100	gift of D.M. Holtzman, Washington University, St. Louis, MO [57]	(HJ6.3)
Neuronal nuclear protein	NeuN	Mouse	1:150	Sigma-Aldrich, St. Louis, MO	MAB377 (A60)
Transmembrane protein 119	TMEM119	Rabbit	1:350	GeneTex, Irvine, CA	GTX134087
Triggering receptor expressed on myeloid cells 2	TREM2	Sheep	1:150	R&D Systems, Minneapolis, MN	AF1729

**Table 2:** List of secondary antibodies used for immunohistochemistry

Antigen	Host	Conjugate	Source	Catalog #
Anti-mouse IgG	Goat	Alexa 594	Jackson Immuno Research Labs, West Grove, PA	115–585-146
Anti-mouse IgG	Goat	Alexa 488	Jackson Immuno Research Labs	115–545-166
Anti-rabbit IgG	Goat	Alexa 488	Jackson Immuno Research Labs	111–545-144
Anti-rabbit IgG	Goat	Alexa 594	Jackson Immuno Research Labs	111–585-144
Anti-rabbit IgG	Donkey	Alexa 488	Jackson Immuno Research Labs	711–545-152
Anti-rabbit IgG	Goat	Biotin	Vector Laboratories, Burlingame, CA	BA-1000
Anti-rat IgG	Goat	Alexa 594	Jackson Immuno Research Labs	112–585-143
Anti-sheep IgG*	Donkey	Alexa 594	Invitrogen, Waltham, MA	A-11016

Working dilution 1:500, except* diluted 1:400

**Table 3:** List of primary and secondary antibodies used for Western immunoblotting

Antigen	Abbreviation	Host	Dilution	Source	Catalog # (Clone)
Beta-actin	β-actin	Mouse	1:10,000	Sigma-Aldrich, St. Louis, MO	A2228 (AC-74)
Cluster of differentiation 230	CD230	Mouse	1:5,000	BioLegend, San Diego, CA	808,001 (6D11)
Glial fibrillary acidic protein	GFAP	Rabbit	1:15,000	Dako/Agilent Technologies Santa Clara, CA	Z0334
Human apolipoprotein E	ApoE	Goat	1:7,000	Meridian Life Science Inc., Memphis, TN	K74180B
Murine apolipoprotein E	ApoE	Mouse	1:5,000	Gift of D.M. Holtzman, Washington University St. Louis, MO [57]	(HJ6.3)
Goat IgG, HRP-linked secondary antibody		Donkey	1:30,000	Santa Cruz Biotechnology Inc., Dallas, TX	Sc-2020
Rabbit IgG F(ab’)2 specific, HRP-linked secondary antibody		Donkey	1:30,000	GE Healthcare Bio-Science Corp., Pittsburgh, PA	NA9340
Mouse IgG F(ab’)2 specific, HRP-linked secondary antibody		Donkey	1:30,000	GE Healthcare Bio-Science Corp., Pittsburgh, PA	NA9310

### Animals

All mouse care and experimental procedures were approved by Institutional Animal Care and Use Committees of the New York University Grossman School of Medicine. *Apoe*^*−/−*^ mice (B6.129P2-Apoe^tm1Unc^/J Stock No: 002052; The Jackson Laboratories, Bar Harbor, ME) [60, 84] and genetic background-matching wild type (WT) mice (C57BL/6J Stock No: 000664) were bred in house. At the age of 8 to 10 weeks male and female mice (~ 50%:50% sex ratio) were intraperitoneally inoculated with a 100 μL of 22L infectious brain homogenate. The 10% (wt/vol) 22L brain homogenate was prepared according to our published protocols from mice killed in the terminal stage of prion disease [5, 6, 56, 59, 65]. Control mice were inoculated with 10% (wt/vol) normal brain homogenate (NBH) prepared from healthy 8 – 10 week-old WT B6 mice. A single batch of 22L and NBH inoculum was used throughout the entire study. Following the inoculation, animals were kept separated by sex in germ free vivarium under institutionally approved animal biosafety level 2 protocol. The mice were exposed to 12-h light/dark cycle and had free access to food and water. Mouse health was assessed bi-weekly following standards of good husbandry practice [7]. Starting from the 10th week post inoculation (wpi) mice were tested once a week using the parallel bar crossing test. This procedure has been validated previously by us and others for reliable detection of the early signs of prion disease [5, 35, 64, 65]. It involves assessment of the mouse's competency to cross a series of parallel bars that are 3 mm in diameter and are placed 7 mm apart by an observer blinded to the animal experimental status. A mouse, which displays an impaired level of activity and/or competency when attempting to cross the parallel bars for three weeks in a row is recognized as symptomatic and the first time it scores positively is in retrospect considered the day of disease onset. To detail the progression of clinical symptoms of the prion disease we also used the scrapie severity score, which is a 15-point scale assigning 0—3 points for each of the following features: arousal, hind-limb-weakness, body posture (kyphosis), gait-abnormalities, and body condition [11, 12, 73]. The scoring system for each feature is based on the following criteria 0 = normal, 1 = subtle, 1.5 = mild, 2 = moderate, 2.5 = advanced 3 = severe. 22L inoculated animals were killed either at 15 wpi when *Apoe*^*−/−*^ and WT mice remain asymptomatic or at 23 wpi when both genotypes display overt symptoms of prion disease. NBH inoculated controls were killed at 23 wpi and they were neurologically asymptomatic. Mice were killed by an intraperitoneal injection of Euthasol (500 μL/kg) (Virbac AH, Inc.; Westlake TX) and transcardially perfused with 10 mM phosphate-buffered saline (PBS) (pH 7.4) with addition of heparin (1,000 units/L). Immediately after the perfusion brains were removed from the skulls. The olfactory bulbs and the cerebellum were cut off and the brains were divided along the longitudinal fissure into left and right hemispheres. The brain cortex and the hippocampus were isolated from the left hemisphere and either used for RNA extraction or immediately flash frozen in a dry ice/methanol cooling bath, and stored at − 80 °C until further analyses. The right brain hemisphere was dissected in the frontal plane at approximately + 1 mm Bregma level. The rostral portion was fixed in 2% phosphate-buffered formalin and embedded in paraffin, while the larger posterior part was fixed in 4% paraformaldehyde in 0.1 M phosphate buffer [PB], pH 7.4 at 4 °C over 7 days, and dehydrated in 2% dimethyl sulfoxide and 20% glycerol in 0.1 M PB, pH 7.4 at 4 °C.

### Histology, immunohistochemistry, and quantitative analyses

The paraffin embedded, rostral portion of the right hemisphere was cut into 8-μm-thick coronal sections, which were stained with hematoxylin and eosin for quantitative analysis of the spongiform lesions. The load of spongiform lesions (% of cross-sectional surface area occupied by the lesions) was quantified in the M1 primary motor cortex. The quantification was done on digitized images of the M1 cortex taken at approximated Bregma levels + 1.0 mm, + 1.2 mm, and + 1.4 mm with deviation not greater than ± 0.1 mm [31, 87]. Image analysis was performed using NIH ImageJ v1.53a software (Bethesda, MD) [68]. The spongiform lesions were negatively thresholded based on the optical density of surrounding brain parenchyma. Vessels, with threshold signal similar to those of spongiform lesions were identified by the presence of ependymal cells and manually edited out. The caudal portion of the right brain hemisphere was cut serially into coronal 40-μm-thick sections using a freezing microtome (Leica Microsystems, Wetzlar, Germany). Serial sections were alternately collected into 10 series, which were kept in the cryoprotectant (30% sucrose, 30% ethylene glycol in 0.1 M PB, pH 7.4) at 4 °C until the immunohistochemistry. Randomly selected series of sections were immunostained against the following antigens: (1) apoE and GFAP in combination, (2) apoE and Iba1 in combination, (3) PrP, (4) GFAP, (5) C3 and GFAP in combination, (6) Iba1 with 4’, 6’-diamidino-2-phenylindole (DAPI), (7) CD68, (8) CD68 and Iba1 in combination, (9) TREM2 and Iba1 in combinations, (10) NeuN, and Iba1, and CD68 in combination, and (11) TMEM119. Prior to immunostaining the sections were incubated in 10 mM sodium citrate and 0.05% Tween 20 (pH 6.0) at 85 °C for 15 min to increase antigen availability [58]. For anti-PrP immunostaining the sections were additionally treated with 98% formic acid at room temperature for 10 min. Non-specific antibody binding was blocked using a blocking solution consisting of 10% normal goat serum and 1% bovine serum albumin (BSA) in 10 mM PBS (pH 7.4) with 0.3% Triton X-100. When the mouse primary antibody was used, 1μL of the mouse on mouse blocking reagent (Vector Laboratories; Burlingame, CA) was added to 1.5 mL of the blocking solution. For TREM2 immunohistochemistry the specific blocking solution consisted of 5% donkey serum in 10 mM PBS (pH 7.4) and 0.3% Triton X-100 [58]. For the protocols utilizing biotinylated secondary antibody the blocking was with 5% BSA in 10 mM PBS (pH 7.4) and 0.3% Triton X-100 followed by Avidin/Biotin Blocking Kit (Vector Laboratories). Detailed list of primary antibodies used in this study and their working dilutions is given in Table 1, while the choice of secondary antibodies in Table 2. For multiple antigen staining procedures a cocktail of primary and secondary antibodies was prepared. The sections were thoroughly washed with 10 mM PBS (pH 7.4) and 0.1% Triton X-100 between each step of immunohistochemistry procedure. Slides were coverslipped using Depex mounting medium (Thermo Fisher Scientific, Waltham, MA). Additional series of sections were stained with cresyl violet to allow for cytoarchitectonic orientation and enumeration of neurons and with Fluoro Jade C (FJC) (Histo-Chem Inc., Jefferson, AR) an anionic florescent dye, with confirmed selective affinity toward degenerating neurons [18, 19].

Images of immunostained sections were digitized as previously described [57, 58] and used for quantitative analysis, which was carried out using NIH ImageJ v1.53a. The load of GFAP^+^ astrocytes, and Iba^+^ and CD68^+^ microglia was quantified in the lateral and the medial parts of the ventral posterior thalamic nucleus (VPN) and in the S1 primary somatosensory cortex, which in the extra-CNS transmitted prion model are affected sequentially. The load of TMEM119^+^ microglial cells was quantified in the S1 primary somatosensory cortex only. The load is defined as the percentage of a cross-sectional area of the given anatomical structure covered by positively-thresholded objects and it was determined across the entire cross-sectional profile of the VPN or the S1 cortex to prevent sampling bias. Three VPN profiles (Bregma -1.4 mm, -1.7 mm, -2.0 mm) and three S1 profiles (Bregma 0 mm, -1.0 mm, -1.8 mm) were analyzed per each brain. Deviation from each pre-set Bregma plane was not greater than ± 0.2 mm. Load values obtained from three profiles were averaged for each brain. We also determined relative expression of C3, CD68, and TREM2 by indexing the C3 load to that of GFAP and the load of CD68 and TREM2 to that of Iba1 in the S1 cortex. To analyze proliferation of microglial cells Iba1^+^/DAPI^+^ cells discernible within the entire optical depth of the section were enumerated in the layer V of the S1 cortex on microphotographs taken under × 40 magnification. Their count was divided by the area of the layer V as it appears on the photograph to obtain a numerical density value. In similar way we enumerated the percentage of NeuN^+^ neurons directly associated with CD68^+^/Iba1^+^ microglia, numerical density of cresyl violet stained neurons, and FJC^+^ neurons in the layer V of the S1 cortex. In addition, we analyzed integrated density of anti-PrP immunostaining on three cross-sectional profiles throughout the S1 cortex.

### Laser scanning confocal microscopy (LSCM) imaging and the apoE CTCF analysis

Randomly selected series of sections from NBH and 22L inoculated WT mice, which were killed at 23 wpi, were double immunostained against apoE and GFAP and against apoE and Iba1 to perform cell-type specific analysis of the apoE protein expression in astrocytes and microglia, respectively. Z stacks of double immunostained astrocytes and microglia in the layer V of the S1 cortex were taken under immersion oil, 63x, and 1.4 N.A. objective and 2 × digital zoom using Zeiss LSM 880 microscope and ZEN Black 2.3 SP1 acquisition software v. 14.0.18.201 (Carl Zeiss AG; Oberkochen, Germany). Z stacks of 0.5-μm-thick serial tomograms with 25% overlap were acquired through the entire thickness of the cell. Images representing the biggest cross-section of an analyzed cell were extracted from confocal Z stacks and contrast enhanced. The corrected total cell fluorescence (CTCF) for the apoE signal within the GFAP^+^ and Iba^+^ astrocytes and microglia was analyzed using NIH ImageJ2 (Fiji) (Bethesda, MD) [66] following our previously published protocols [59], respectively. Additionally we used LSCM to detail expression of C3 in activated astrocytes and CD68 and TREM2 in activated microglia. Z stack images of C3/GFAP double immunostained astrocytes and CD68/Iba1 and TREM2/Iba1 double immunostained microglia were taken in the layer V of the S1 cortex using LSCM equipment and conditions described above for the analysis of apoE in these cells.

Three-dimensional rendering of NeuN immunostained neurons opsonized by CD68^+^/Iba1^+^ microglia was done from Z stacks of 0.2-μm-thick serial tomograms with 80% overlap, which were taken under immersion oil, 63x, and 1.4 N.A. objective and 2.5 × digital zoom. The three-dimensional rendering was performed using Imaris v9.2 imaging software (Bitplane AG, Zurich, Switzerland).

### Quantitative western blot analysis of brain proteins

Samples of the brain cortex were removed from − 80 °C storage and homogenized 1:10 (wt/vol) at 4 °C in a buffer consisting of 20 mM Tris–HCL (pH 7.4), 250 mM sucrose, 1 mM egtazic acid, 1 mM ethylenediaminetetraacetic acid and 10 μg/mL of a Complete Protease Inhibitor Cocktail (Roche Life Science, Indianapolis, IN). The homogenization was a three-step procedure, which included manual trituration in a pestle grinder, passing the triturated tissue through a 28-gauge needle, and final sonication. Cellular debris were cleared by 3-min. centrifugation at 10,000 × g and 4 °C. Protein concentration in the resulting supernatant was measured by bicinchoninic acid (BCA) method using Pierce™ BCA Protein Assay Kit (Thermo Fisher Scientific), according to the manufacturer provided manual. Aliquots of brain homogenate containing either 20 μg of the total protein for apoE detection or 5 μg of the total protein for GFAP and total PrP detection were mixed with sample buffer containing β-mercaptoethanol, boiled for 5 min. and resolved using 10% sodium dodecyl sulphate–polyacrylamide gel electrophoresis (SDS-PAGE) followed by electroblotting onto nitrocellulose membranes. The membranes were blocked overnight at 4 °C with 5% nonfat milk (or 5% soy milk for apoE) in 10 mM tris-buffered saline (pH 7.4) and 0.1% Tween 20 and then incubated with the primary antibodies, which are listed along with their working dilutions in Table 3. The antigen–antibody complexes were detected using horseradish peroxidase (HRP) conjugated sheep anti-mouse or donkey anti-rabbit secondary antibodies detailed in Table 3 and visualized using SuperSignal West Pico PLUS Chemiluminescent Substrates (Thermo Fisher Scientific) by apposing the membranes to HyBlot CL® autoradiography film (Thomas Scientific, LLC, Swedesboro, NJ). Resulting autoradiograph were digitized into a 600 dpi TIFF files as previously described [6, 47, 65]. Equal protein load across the samples was verified by stripping the nitrocellulose membranes using Restore™ Western Blot Stripping Buffer (Thermo Fisher Scientific) and re-probing them against β-actin (Table 3) with HRP conjugated sheep anti-mouse as the secondary antibody.

To quantify the PrP^Sc^ level, aliquots of brain homogenate containing 10 µg of total protein were digested with proteinase K (PK) (Roche Life Science) (1:10 enzyme to protein weight ratio [17, 18]) in 20 μL volume at 37 °C for 30 min. PK activity was stopped by adding 4 μL of 100 mM phenylmethylsulfonyl fluoride. Digested samples were centrifuged for 45 min at 20,000 × g and 4 °C. The supernatant was discarded and the pellets were resuspended in 15 µl PBS and 15 µl sample buffer containing β-mercaptoethanol, boiled for 5 min, resolved using 12.5% SDS-PAGE, and electroblotted onto nitrocellulose membranes, which were overnight blocked with 5% nonfat milk at 4 °C. PK-resistant PrP^Sc^ was detected using anti-CD230 antibody (clone 6D11) [6, 65] (Table 3) followed by HRP conjugated sheep anti-mouse secondary antibody.

Digitized autoradiographs were densitometrically analyzed using NIH ImageJ v1.53a. Levels of total PrP and PrP^Sc^ were quantified by measuring protein band optical densities corresponding to non-, mono- and diglycosylated PrP forms, level of GFAP by measuring optical densities of its 55 and 48 kDa bands, and levels apoE and β-actin by measuring optical densities of their single bands.

### Analysis of PrP^Sc^ in apoE4 treated 22L/N2A cells

N2A line (line number CCL 131 [American Type Culture Collection; Manassas, VA]) was infected with the 22L strain as detailed in our previous publications [56, 59]. N2A/22L cells were maintained in Dulbecco's modified Eagle's medium (DMEM) supplemented with 10% heat inactivated FBS, penicillin (100 units/mL), and streptomycin (100 µg/mL) and serially passaged. The fifth passage was treated with conditioned media prepared from immortalized astrocytes expressing natively lipidated human apoE4 or those from *Apoe*^*−/−*^ astrocytes [53]. Prior to harvesting the media, 80–85% confluent astrocytic culture was grown in DMEM/F-12 supplemented with 1 mM sodium pyruvate, N-2, and 3 mM of 25-hydroxycholesterol for 48 h. Production of apoE4 lipidated complexes was confirmed by resolving media samples on native and SDS PAGE [53], (Additional file 1: Fig S4a, b) followed by western transfer and immunoblotting with anti-human apoE antibody (Table 3). Astrocytic media were tenfold concentrated using Amicon Ultra centrifugal filter units (10 kDa MWCO) (MilliporeSigma, Burlington, MA), which were primed with 0.1 mg/ml of bovine serum albumin to prevent apoE sticking into the filter membranes. At the commencement of the experiment N2A/22L cells were 60% confluent. Concentrated astrocytic media were mixed with N2A/22L growth media 1:9 vol/vol. N2A/22L cells were cultured for continuous 96 h. replacing the apoE containing medium every 12 h. At the completion of the experiment 95–98% confluent N2A/22L cells were lysed with the ice cold lysis buffer (50 mM Tris–HCL [pH 7.5], 150 mM NaCl, 0.5% triton X-100, 0.5% sodium deoxycholate, and Complete Protease Inhibitor Cocktail) and centrifuged for 3 min. at 10,000 × g to remove cell debris. Protein concentration in the resulting supernatant was determined using BCA assay. Aliquots containing 100 μg of total protein were titrated to achieve 1 μg: 1 μL protein concentration and subjected to PK digestion as previously described [56, 59]. Samples of PK digested and non-digested N2A/22L cell lysates were subjected to SDS-PAGE, western transfer, and PrP immunoblotting using anti-CD230 antibody (clone 6D11) [6, 65] (Table 3) as described above.

### Quantitative analysis of cytokines

Mouse Inflammatory Cytokines Multi-Analyte ELISArray (Qiagen Sciences Inc., Germantown, MD) was used to compare levels of cytokines in the brain homogenate of 22L *Apoe*^−/−^ and 22L WT mice killed at 23wpi. The assay detects 12 cytokines: IL-1α, IL-1β, IL-2, IL-4, IL-6, IL-10, IL-12, IL-17A, IFN-γ, TNF-α, G-CSF, and GM-CSF. Samples containing equal volume of brain homogenate, which was prepared analogously to that used for Western blotting, were tenfold diluted in the sample buffer included with the kit. The assay was carried out following manufacturer provided manual. Optic densities (OD) of ELISA readouts were measured using the Epoch Microplate Spectrophotometer (BioTek Instruments, Winooski, VT). Results were reported as the fold change of OD values in the 22L *Apoe*^*−/−*^ mice relative to the average value in 22L WT mice. Subsequently, we quantified the level of IL-1β in NBH and 22L inoculated *Apoe*^*−/−*^ and WT mice killed at 15 and 23 wpi using Mouse IL-1β ELISA Kit (R&D Systems, Minneapolis, MN). Samples of brain homogenate were tenfold diluted in 10 mM PBS (pH 7.4) containing 1% highly purified BSA (Fraction V, RIA and ELISA Grade; Millipore Corp., Billerica MA). The assay was carried out following manufacturer provided manual. Optic densities of ELISA readouts were converted to the actual concentrations of IL-1β based on the standard curve prepared from the standards included in the kit. The standard curve was generated using a nonlinear curve-fitting algorithm in GraphPad Prism v 8.4.3 (GraphPad Software, San Diego, CA). Final IL-1β concentration was reported in pg/mL considering sample dilution.

### mRNA analysis by nanoStringTM nCounter®

Total RNA was extracted from the brain cortex immediately after the perfusion using RNeasy Mini Kit (Qiagen Sciences Inc.) and treated with 2 U of DNAse I (Qiagen Sciences Inc.). Purity and integrity of the isolated RNA was determined using 2100 Bioanalyzer (Agilent Technologies Inc., Santa Clara, CA) and its concentration was measured using NanoDrop™ 2000 spectrophotometer (Thermo Fisher Scientific). One hundred nanograms of total RNA was analyzed using the nCounter® Mouse Glial Profiling assay (NanoString Technologies, Inc., Seattle, WA), which covers 770 gene targets specific to glial cell biology, homeostasis, activation and neuroinflammatory pathways along with a set of 13 internal reference genes. nCounter chips were processed by the Genome Technology Center of the NYU Langone School of Medicine using the nCounter® MAX Analysis System (NanoString Technologies Inc.). Gene expression was analyzed using nSolver™ Analysis Software v4.0 (NanoString Technologies Inc.) following manufacturer provided manual and adjusted based on the expression level of the reference genes. Heatmaps of gene expression data along with hierarchical cluster analysis were generate using the nSolver™ software for genes, which had a minimum of 25 counts per brain. Glial transcript was analyzed in NBH and 22L inoculated WT and *Apoe*^*−/−*^ mice, which were killed at 23 wpi. There were 3 mice per group except for NBH WT mice where there were 2 mice per group. Magnitude of change in Nanostring nCounter values in 22L WT and 22L *Apoe*^*−/−*^ mice also was analyzed as a fold difference relative to the average values of pooled NBH inoculated WT and *Apoe*^*−/−*^ mice, as these two groups showed no statistically significant differences.

### Quantitative Real Time PCR (qRT-PCR) analysis

Five-hundred nanograms of RNA, extracted from the brain cortex as described above, were reverse transcribed into cDNA with the help of the iScript™ Advanced cDNA Synthesis Kit (Bio-Rad Laboratories, Hercules, CA). qRT-PCR was performed using SYBR® Green JumpStart™ Taq ReadyMix™ on a CFX96™ Real-Time System (Bio-Rad Laboratories). The sequences of primers for target genes and for *Gapdh*, which was used as the reference gene are given in Table 4. Amplification efficiency for each primer pair was checked by calculating the regression slope between the cDNA dilution log values and the averaged threshold cycle (C_T_) value obtained for each cDNA dilution. Expression of target genes *C3, Gfap, P2ry12, Tmem119, *and *Trem2* was compared across the experimental groups by statistical analysis of ΔC_T_ values [13]. Each ΔC_T_ value represents a difference between the replicate C_T_ values of a target gene and the replicate C_T_ value for *Gapdh*, which were amplified in parallel for an individual animal. Reported in text fold-change in gene expression was calculated based on the 2^−ΔΔCT^ principle [50].

**Table 4:** List of primers used for qRT-PCR

Gene Name	Forward Primer Sequence (5′–3′)	Reverse Primer Sequence (5′–3′)
*C3*	CGCAACGAACAGGTGGAGATCA	CTGGAAGTAGCGATTCTTGGCG
*Gapdh*	AGGTCGGTGTGAACGGATTTG	TGTAGACCATGTAGTTGAGGTCA
*Gfap*	GGCGCTCAATGCTGGCTTCA	TCTGCCTCCAGCCTCAGGTT
*P2ry12*	CATTGACCGCTACCTGAAGACC	GCCTCCTGTTGGTGAGAATCATG
*Tmem119*	CCTTCACCCAGAGCTGGTTC	GGCTACATCCTCCAGGAAGG
*Trem2*	CTACCAGTGTCAGAGTCTCCGA	CCTCGAAACTCGATGACTCCTC

### Data analysis and statistical analysis

The length of prion disease incubation is defined as the number of days between the inoculation and occurrence of the first clinical symptoms. It was analyzed using the Kaplan–Meier estimator and the differences between groups were compared using log–rank test. The scrapie severity score between 22L infected WT and *Apoe*^*−/−*^ mice was compared using two-way analysis of variance (ANOVA). Data sets for various neuropathological and biochemical outcome measures and nanoStringTM nCounter® values were first assessed for normality using Shapiro–Wilk test. Differences across three or more data sets with normal distribution were compared by one-way ANOVA followed by Holm-Sidak’s post hoc test. Differences between two data sets were compared using unpaired, two-tailed *t*-test with Welch’s correction. Statistical analysis was performed using GraphPad Prism v 8.4.3 (GraphPad Software). All data in the manuscript are presented as the mean and the standard error of the mean (SEM).

## Results

### Prion infection increases brain apoE level and causes cell-type shift in the apoE expression

**Fig. 1:**
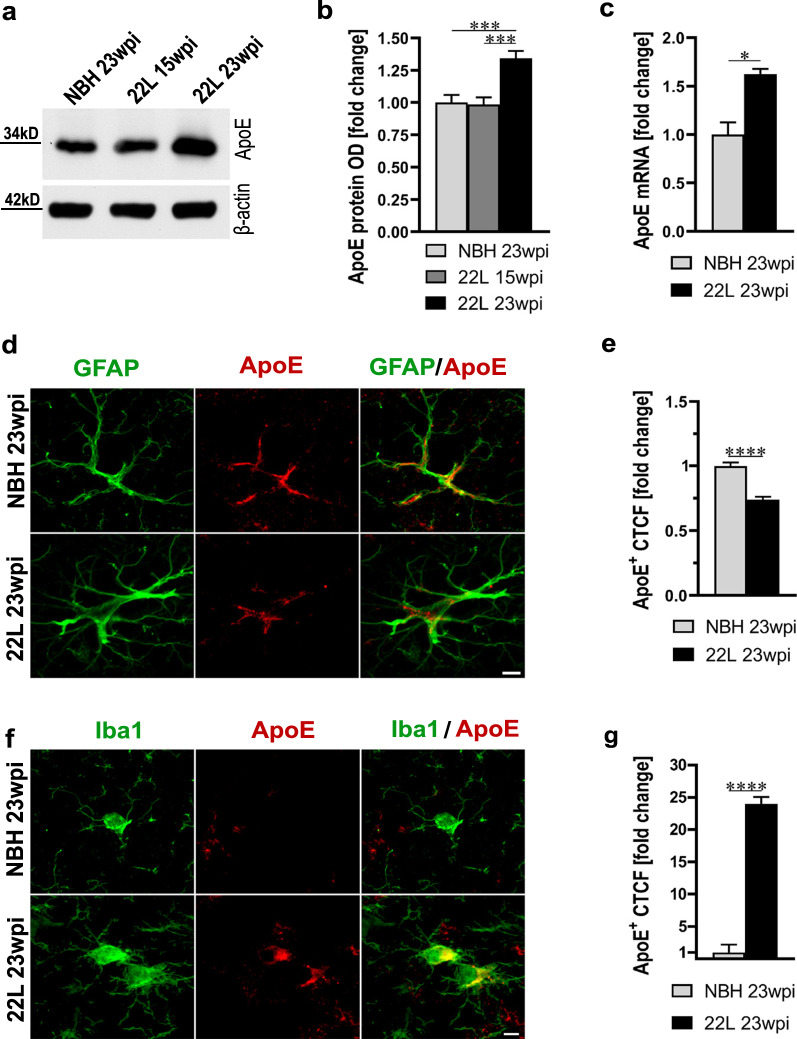
Prion infection of B6 WT mice is associated with upregulation of brain apoE level and cell-type shift in the apoE expression.** a** Shown is immunoblot analysis of the apoE protein level in the brain cortex of B6 mice inoculated with NBH or 22L mouse adapted scrapie strain at 15 and 23 wpi with β-actin as the loading control. **b** Densitometric quantification of apoE protein band optical densities (OD). Values represent fold change relative to those in the NBH group (n = 8–10 mice per group). **c** Shown is analysis of *Apoe* mRNA in the brain cortex. Values represent fold change relative to those in the NBH group (n = 3 mice per group). 22L inoculated mice show significant increase both in apoE protein and *Apoe* RNA level at 23 wpi. **d** and **f** Representative LSCM images of astrocytes and microglia double immunostained for apoE and GFAP or Iba1 from NBH and 22L inoculated B6 mice at 23 wpi, respectively. **e** and** g** Quantification of apoE CTCF in double immunostained astrocytes and microglia, respectively; revealing reduced apoE expression in astrocytes and increased apoE expression in microglia in 22L mice. Values are expressed relative to those in NBH group (n = 65–75 cells per group). **b** *p* < 0.0001 (ANOVA); ***p < 0.001 (Holm’s-Sidak’s post hoc test). **c**, **e**, and **g** *p < 0.05, and ****p < 0.0001 (two-tailed *t*-test with Welch’s correction). Scale bar: 5 μm in **d** and **f**. All numerical values represent mean + SEM

We first compared the total brain apoE level and its expression by astrocytes and microglia in wild-type (WT) B6 mice, which were intraperitoneally inoculated with 22L mouse adapted scrapie strain or NBH. 22L inoculated B6 mice were killed at 15 and 23 week post inoculation (wpi), when animals remain neurologically asymptomatic or show overt clinical symptoms of prion disease, respectively. NBH controls were killed at 23 wpi and they were clinically asymptomatic. At 15 wpi the apoE protein level in the brain cortex of 22L inoculated mice is comparable to that of NBH inoculated controls; however, at 23 wpi 22L inoculated mice show 1.3-fold increase in the level of apoE protein (*p* < 0.001) and 1.6-fold increase in the level of apoE mRNA (*p* < 0.05) relative to NBH controls, respectively (Fig. 1a–c). Cell-type specific determination of apoE expression was performed in the brain cortex by apoE CTCF analysis in apoE/GFAP and apoE/Iba1 double immunostained astrocytes and microglia, respectively. It shows 0.74-fold decrease in the apoE signal in astrocytes (*p* < 0.0001) (Fig. 1d, e) and 24-fold increase in the apoE signal in microglia (*p* < 0.0001) (Fig. 1f, g) in 22L mice relative to NBH controls.

### Absence of apoE shortens prion disease incubation time and increases pathology burden

**Fig. 2:**
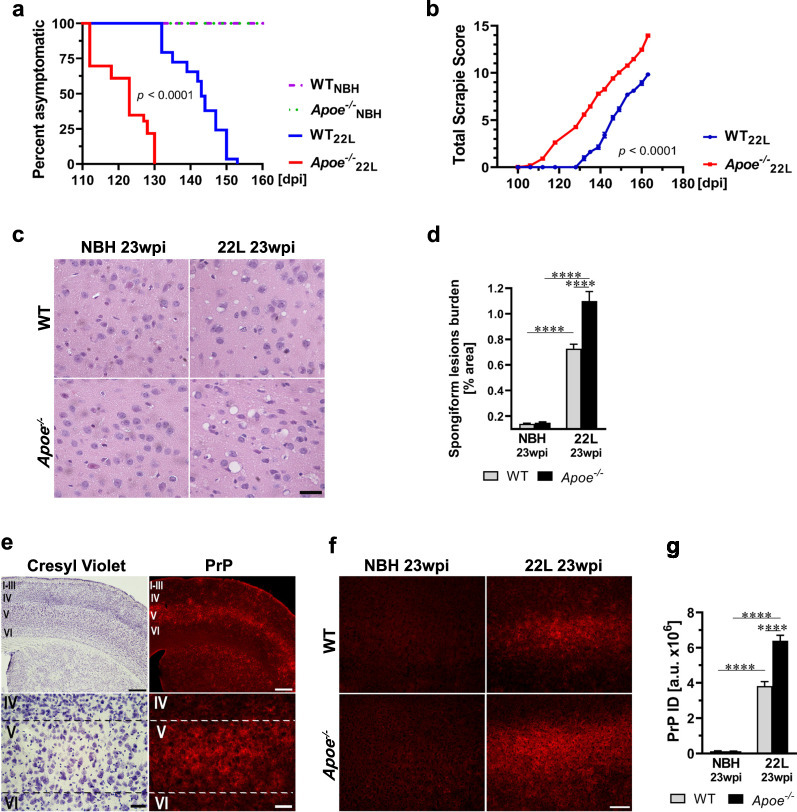
*Apoe*^*−/−*^ mice have shorter incubation time and exaggerated prion pathology. **a** Kaplan–Meier estimator of the incubation time in 22L and NBH inoculated WT and *Apoe*^*−/−*^ mice. The x-axis denotes days post inoculation (dpi) while the y-axis a percent of animals, which remain asymptomatic from the initial count of 29 22L inoculated WT mice, 23 22L inoculated *Apoe*^*−/−*^ mice and 22–23 NBH inoculated WT and *Apoe*^*−/−*^ mice each. **b** Shown is the Total Scrapie Score longitudinally assessed in 22L inoculated WT and *Apoe*^*−/−*^ mice from 12 wpi. Values represent mean ± SEM from 11 to 12 mice per group. **c** Representative microphotographs of the M1 motor cortex (Bregma + 1.5 mm) stained with hematoxylin and eosin to show differences in the load of spongiform pathology. **d** Quantification of the spongiform lesion burden in the M1 cortex representing mean values + SEM from 6 to 7 mice per group.** e** Microphotographs of the S1 somatosensory cortex (Bregma + 0.0 mm) in 22L WT mice (23 wpi) stained with cresyl violet for cytoarchitectonic orientation or immunostained against PrP to assess distribution of PrP deposition, which shows preponderance for accumulation in the layer V. **f** Representative microphotographs of anti-PrP immunostaining in the S1 cortex in various animal groups and **g** Integrated density (ID) quantification of the anti-PrP signal in the S1 cortex. Shown are means + SEM from 5 to 7 mice per group. **a** *p* < 0.0001 denotes the significance between 22L *Apoe*^*−/−*^ and WT groups. Differences between 22L *Apoe*^*−/−*^ and NBH *Apoe*^*−/−*^ and between 22L WT and NBH WT groups are not shown in the graph but they also are significant at *p* < 0.0001 (Log-Rank test). The difference between NBH WT and NBH *Apoe*^*−/−*^ is not statistically significant. **b** *p* < 0.0001 (2-way ANOVA). **d, g** *p* < 0.0001 (ANOVA); ****p < 0.0001 (Holm’s-Sidak’s post hoc test). Scale bars: 30 μm in **c**, 350 μm (upper panel) and 40 μm (lower panel) in **e**, and 100 μm in **f**

The length of the prion disease incubation period was determined by longitudinal locomotor behavioral testing using the parallel bar crossing test [5, 64, 65]. Starting from 10 wpi all mice were tested weekly by an observer blinded to their genotype and the type of inoculum they received. Mice, which display gross motor incompetence for three weeks in a row are considered symptomatic and the first day they score positively is considered in retrospect the disease onset. Kaplan-Meyer estimator was used to analyze differences in the prion incubation time across the animal groups. 22L inoculated *Apoe*^−/−^ mice have 20 days shorter median incubation time than 22L inoculated WT mice (*p* < 0.0001) (Fig. 2a). No neurological symptoms were identified in either NBH inoculated WT or *Apoe*^−/−^ mice by 23 wpi. To assure the effect of apoE absence on the prion incubation time is not strain specific we also inoculated mice with the ME7 strain and likewise noticed statistically significant difference in the median disease incubation period between ME7 WT mice and ME7 *Apoe*^−/−^ mice, which was 26 days shorter in the latter group (*p* < 0.0001) (Additional file 1: Fig. S1). Separate cohorts of 22L inoculated WT and *Apoe*^−/−^ mice also were subjected to longitudinal assessment of the Total Scrapie Score, which gives a quantitative measure of disease progression and severity. The individual components of the Scrapie Score were assessed from 12 wpi onward by two independent examiners who were blinded to the animal genotype (Additional file 1: Fig. S2a-e). As shown in Fig. 2b in 22L *Apoe*^−/−^ mice the symptoms occur earlier and their course is more aggressive (*p* < 0.0001). At 23 wpi when mice are killed the Total Scrapie Score in 22L *Apoe*^−/−^ mice is by 4.1 points higher than that in 22L WT mice.

Detailed neuropathological analysis was carried out in symptomatic animals killed at 23 wpi. In 22L inoculated *Apoe*^*−/−*^ mice the load of spongiform lesions in the M1 primary motor cortex is 1.5-fold higher (*p* < 0.0001) (Fig. 2c, d) and the integrated density of immunostained PrP deposits in the brain cortex is 1.7-fold higher (*p* < 0.0001) (Fig. 2e–g) relative to those in 22L inoculated WT mice. PrP deposition shows a particular predilection to the layer V of the neocortex (Fig. 2e, Additional file 1: Fig. S3), the thalamus (Additional file 1: Fig. S3), the entorhinal cortex, the globus pallidus, and the amygdala (not shown). In affected structures, PrP forms conspicuous clusters (Additional file 1: Fig. S3), which tend to merge covering the entire cross-sectional area of the structure. There are no anatomical differences in the PrP distribution between 22L WT and 22L *Apoe*^*−/−*^ mice. The main dissimilarity is that in the former group the intensity of anti-PrP immunostaining is more pronounced and the PrP clusters appear more conspicuous (Additional file 1: Fig. S3).

**Fig. 3:**
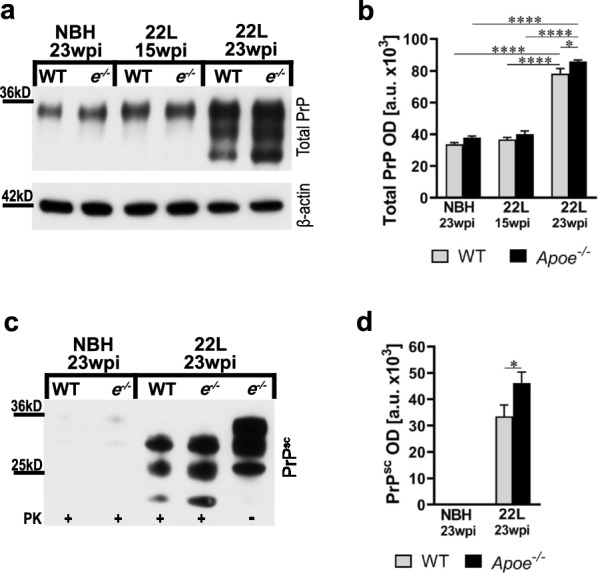
*Apoe*^*−/−*^ mice accumulate more PrP^Sc^. **a** and** c** Immunoblot analysis of the total PrP protein and that of proteinase K (PK) resistant PrP^Sc^ in the brain cortex in B6 WT and *Apoe*^*−/−*^ (*e*^*−/−*^) lines, respectively. Also included are β-actin as the loading control in **a** and PK digestion control (last lane) in **c**. **b** and **d** Densitometric quantification of total PrP and PrP^Sc^ protein band optical densities (OD). Values represent mean + SEM from 8 to 10 mice per group. **b** *p* < 0.0001 (ANOVA); *p < 0.05, and ****p < 0.0001 (Holm’s-Sidak’s post hoc test). **d** *p < 0.05 (two-tailed *t*-test with Welch’s correction)

In parallel, we determined changes in the total PrP protein level and that of PK-resistant PrP^Sc^ in the brain cortex homogenate at 23 wpi by quantitative WB densitometry. Total PrP level is 1.1-fold increased (*p* < 0.05) (Fig. 3a, b) and PK-resistant PrP^Sc^ level is 1.4-fold increased (*p* < 0.05) (Fig. 3c, d) in 22L inoculated *Apoe*^−/−^ mice relative to 22L inoculated WT animals, respectively. To determine whether apoE may directly affect PrP^Sc^ replication we treated 22L infected N2A cells with natively lipidated apoE complexes derived from astrocytes expressing human apoE4 or with control media from *Apoe*^*−/−*^ astrocytes (Additional file 1: Fig. S4a, b). This supplementary experiment showed no apparent effect of apoE presence or absence on the total cellular PrP level or the level of PrP^Sc^ in PrP^Sc^ replicating N2A/22L cells (Additional file 1: Fig. S4c, d).

### Lack of apoE promotes astrogliosis and the induction of A1 neurotoxic phenotype

**Fig. 4:**
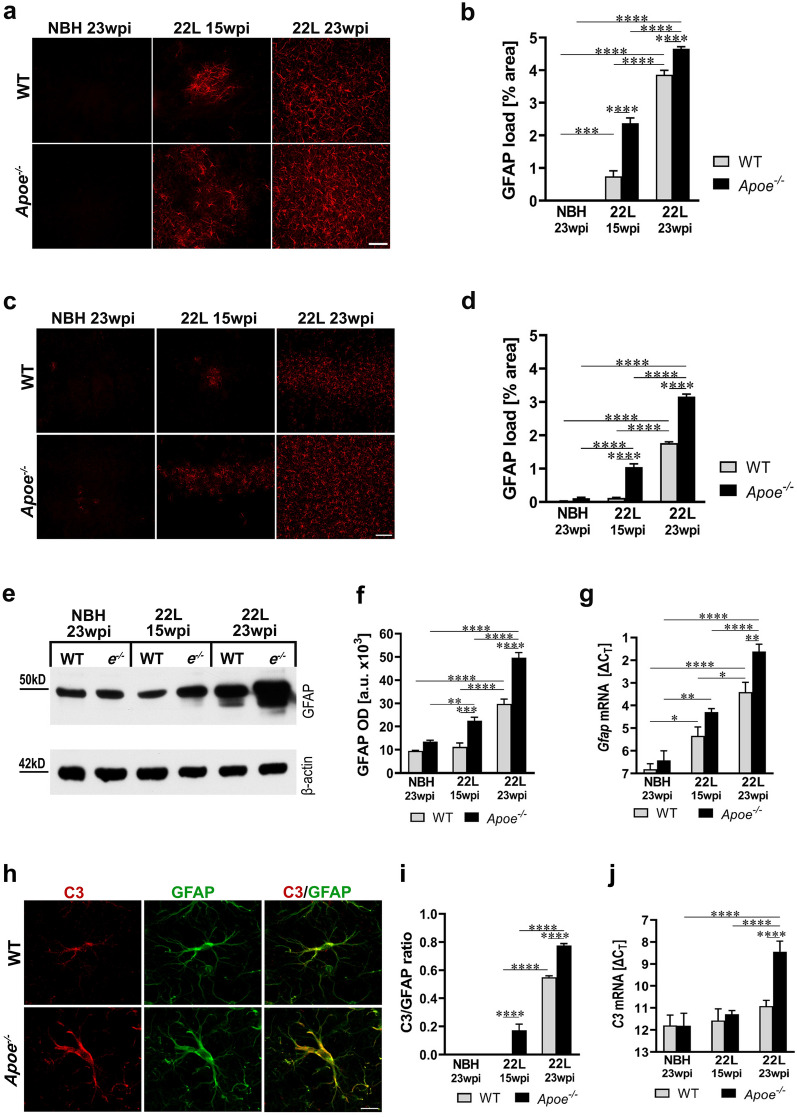
Aggravation of astrogliosis in prion infected *Apoe*^*−/−*^ mice. **a** and **c** Shown are representative microphotographs of coronal cross-sections through the VPN thalamic nucleus and the S1 primary somatosensory cortex immunostained against GFAP from indicated animal groups, respectively. **b** and **d** Quantitative analysis of GFAP^+^ load in the VPN and the S1 cortex, respectively (n = 8–12 mice/group). **e** Immunoblot analysis of GFAP protein level in the brain cortex with β-actin as the loading control and **f** densitometric quantification of GFAP protein band optical densities (OD) (n = 7–12 mice/group). **g** Analysis of *Gfap* mRNA level. The qRT-PCR results are presented as the ΔC_T_ values (n = 3–11 mice/group). In *Apoe*^*−/−*^ mice astrogliosis appears earlier in the course of prion pathology and it is more pronounced than in WT mice. **h** Representative LSCM images of C3/GFAP double immunostained astrocytes in 22L WT and 22L *Apoe*^*−/−*^ mice at 23 wpi. **i** Quantitative analysis of C3/GFAP ratio in the S1 cortex (n = 8–11 mice/group). **j** Analysis of *C3* mRNA level. The qRT-PCR results represent the ΔC_T_ values (n = 3–11 mice/group). There is a marked upregulation of C3 expression during prion infection, which is significantly greater in *Apoe*^*−/−*^ mice. **b**, **d**, **f**, **g, i,** and **j** *p* < 0.0001 (ANOVA); *p < 0.05. **p < 0.01, ***p < 0.001, and ****p < 0.0001 (Holm’s-Sidak’s post hoc test). All numerical values represent mean + SEM. Scale bars: 50 μm in **a**, 100 μm in **c**, and 10 μm in **h**

Astrogliosis and upregulation of GFAP level are hallmarks of prion pathology. We quantified the load of reactive astrocytes (defined as the % of cross-sectional area with GFAP^+^ immunostaining) in the ventral posterior thalamic nucleus (VPN) and in the S1 primary somatosensory cortex, as the thalamus and the brain cortex are affected in sequential order in the intraperitoneal prion inoculation model. In 22L *Apoe*^−/−^ mice, astrogliosis occurs earlier and it is more robust compared to 22L WT mice. At 15 wpi both 22L WT and 22L *Apoe*^−/−^ mice show significant increase in the GFAP^+^ load in the VPN compared to their genotype-matched NBH controls (Fig. 4a, b) but only 22L *Apoe*^−/−^ mice show significantly elevated GFAP^+^ load in the S1 cortex (Fig. 4c, d). At 23 wpi both 22L WT and 22L *Apoe*^−/−^ mice have significantly increased GFAP^+^ load in the VPN and in the S1 cortex over their NBH controls. At either time point the GFAP^+^ load in 22L *Apoe*^−/−^ mice is higher than that in 22L WT mice. At 15 wpi it is 3.2-fold higher in the VPN (*p* < 0.0001) and 8.4-fold higher in the S1 cortex (*p* < 0.0001) relative to 22L WT mice, while at 23 wpi it is 1.2-fold (*p* < 0.0001) and 1.7-fold (*p* < 0.0001) higher in the respective structures. Consistently, there are changes in the level of GFAP protein in the brain cortex revealed by quantitative WB. At 15 wpi only 22L *Apoe*^−/−^ mice show significant increase in the GFAP protein level, while at 23 wpi it is significantly increased both in 22L WT and 22L *Apoe*^−/−^ mice relative to their NBH controls, (Fig. 4e, f). At 15 wpi and 23 wpi the GFAP level in 22L *Apoe*^−/−^ mice is twofold (*p* < 0.001) and 1.7-fold (*p* < 0.0001) higher relative to that in 22L WT mice, respectively. Commensurate changes in the *Gfap* mRNA level across the experimental groups additionally were confirmed by qRT-PCR (Fig. 4g). There are no significant differences in the GFAP load in the VPN and in the S1 cortex and in the level of GFAP protein or in the *Gfap* mRNA level between NBH inoculated *Apoe*^−/−^ and WT mice (Fig. 4a–g).

To gain insight into the profile of astrocyte activation we analyzed expression of the complement component 3 (C3) protein, which is a histological marker of A1 (neurotoxic) astrocyte phenotype [38, 49]. A robust expression of C3 protein in cortical astrocytes already is seen in 22L *Apoe*^−/−^ mice at 15 wpi but not in 22L WT mice (Additional file 1: Fig. S5). At 23 wpi cortical astrocytes both in 22L *Apoe*^−/−^ and 22L WT mice highly express C3 protein, yet the C3/GFAP ratio is 1.5-fold higher in 22L *Apoe*^−/−^ mice relative to 22L WT mice (*p* < 0.0001) (Fig. 4, h, i). Also at 23 wpi *C3* expression calculated from qRT-PCR data is 7.4-fold higher in 22L *Apoe*^*−/−*^ mice than that in 22L WT mice (*p* < 0.0001) (Fig. 4j). C3^+^ astrocytes are undetectable in NBH inoculated WT and *Apoe*^−/−^ mice (Additional file 1: Fig. S5).

**Fig. 5:**
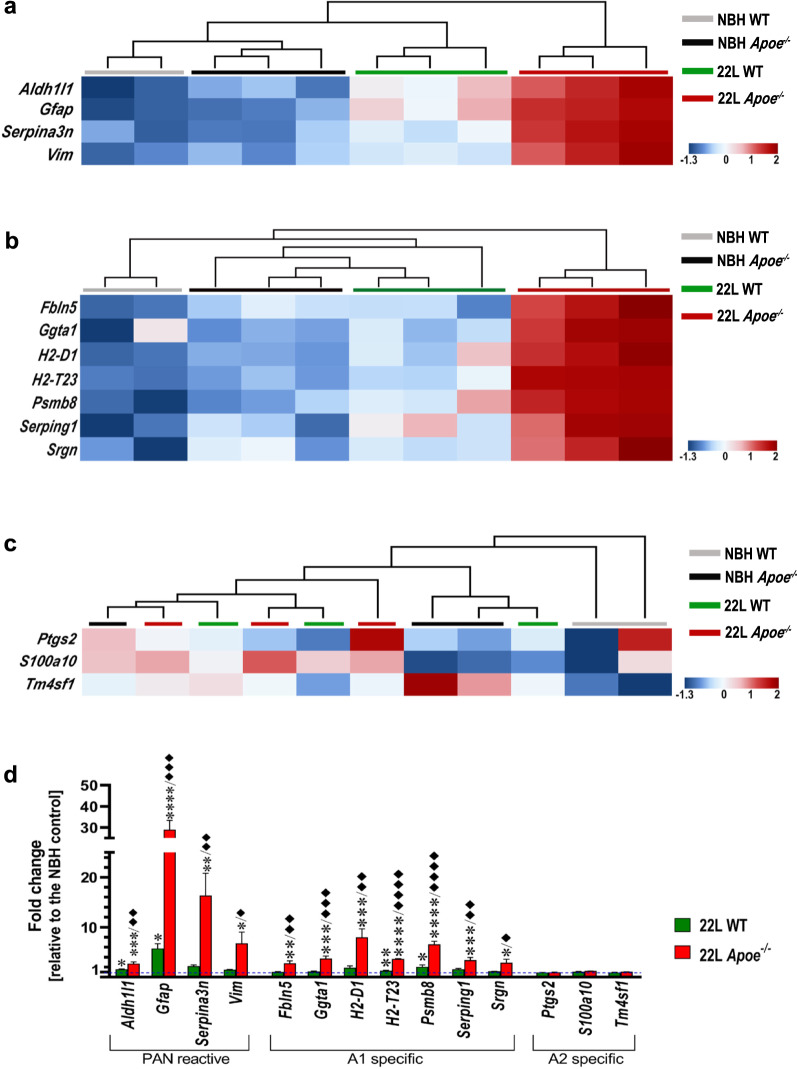
Analysis of astrocytic activation marker expression demonstrates aggravation of A1 astrocyte phenotype in prion infected *Apoe*^*−/−*^ mice. Shown are transcript heatmaps of nanoStringTM nCounter® expression data from NBH and 22L inoculated WT and *Apoe*^*−/−*^ mice, which were killed at 23 wpi. Reactive astrocyte specific transcripts are split into **a** PAN–specific, **b** A1–specific, and **c** A2 -specific cassettes. Hierarchical cluster analysis shows strong separation of 22L *Apoe*^*−/−*^ mice from other experimental groups both for PAN- and A1- specific cassettes. The 22L WT mouse cluster separates from NBH inoculated WT and *Apoe*^*−/−*^ mice within the PAN-reactive cassette but not within A1- specific cassette. No clustering within experimental groups is seen for A2 -specific cassette markers. **d** Fold change of nanoStringTM nCounter® values for PAN- A1-, and A2-reactive astrocyte markers in 22L WT and 22L *Apoe*^−/−^ mice (n = 3 mice/group) relative to those in averaged NBH controls (n = 5 mice/group) at 23 wpi. **d** *p* < 0.01 to *p* < 0.0001 (ANOVA) analyzed separately for each gene from the PAN–and A1-reactive cassettes; *p < 0.05 **p < 0.01 ***p < 0.001, and ****p < 0.0001 22L *Apoe*^−/−^ or 22L WT vs averaged NBH controls; ^♦^*p* < 0.05, ^♦♦^*p* < 0.01, ^♦♦♦^*p* < 0.001, and ^♦♦♦♦^*p* < 0.0001 22L *Apoe*^−/−^ vs 22L WT (Holm’s-Sidak’s post hoc test following significant ANOVA). ANOVA for any of A2-specific cassette gene was not significant. All numerical values represent mean + SEM

Transcriptomic profile of astrocytes was characterized using the nanoStringTM nCounter® technology in animals killed at 23 wpi. Hierarchical cluster analysis of astrocytic transcript shows strong clustering of 22L *Apoe*^*−/−*^ animals and their separation from other experimental groups both for PAN-reactive and A1-reactive cassettes, while 22L WT mice cluster together and separate from other groups only for the PAN-reactive but not for the A1-reactive cassette (Fig. 5a, b). Direct comparison of the nCounter® values shows significant upregulation of the following PAN-reactive markers in 22L *Apoe*^−/−^ mice relative to the averaged NBH controls: *Aldh1l1* (2.7-fold*, p* < 0.001), *Gfap* (29.0-fold*, p* < 0.0001)*, Serpina3n* (16.4-fold*, p* < 0.01)*,* and *Vim* (6.8-fold*, p* < 0.05) (Fig. 5d). In 22L WT mice a significant increase concerns only *Aldh1l1* (1.6-fold*, p* < 0.05), and *Gfap* (5.8-fold*, p* < 0.05)*.* Numerous A1 markers also are significantly elevated in 22L *Apoe*^−/−^ mice relative to the averaged NBH controls: *Fbln5* (2.8-fold*, p* < 0.01), *Ggta1* (3.7-fold*, p* < 0.001)*, H2-D1* (8.0-fold*, p* < 0.001)*, H2-T23* (3.7-fold*, p* < 0.0001)*, Psmb8* (6.6-fold*, p* < 0.0001)*, Serping1* (3.4-fold*, p* < 0.001) and* Srgn* (2.9-fold*, p* < 0.05) (Fig. 5d). Likewise, modest upregulation of these markers is observed in 22L WT mice but reaches statistical significance only for *H2-T23* (1.3-fold*, p* < 0.01)*,* and *Psmb8* (2.1-fold*, p* < 0.05). In direct comparison of nCounter® values between 22L *Apoe*^−/−^ and 22L WT mice, there is a statistical post-hoc significance for all PAN- and A1-reactive markers with the former group showing numerical advantage (Fig. 5d). A2-reactive cassette markers demonstrate no specific clustering at group level (Fig. 5c) or statistically significant increase in either 22L WT or 22L *Apoe*^−/−^ group relative to the averaged NBH controls (Fig. 5d).

### Absence of apoE exaggerates microglia activation and their MGnD transition

**Fig. 6:**
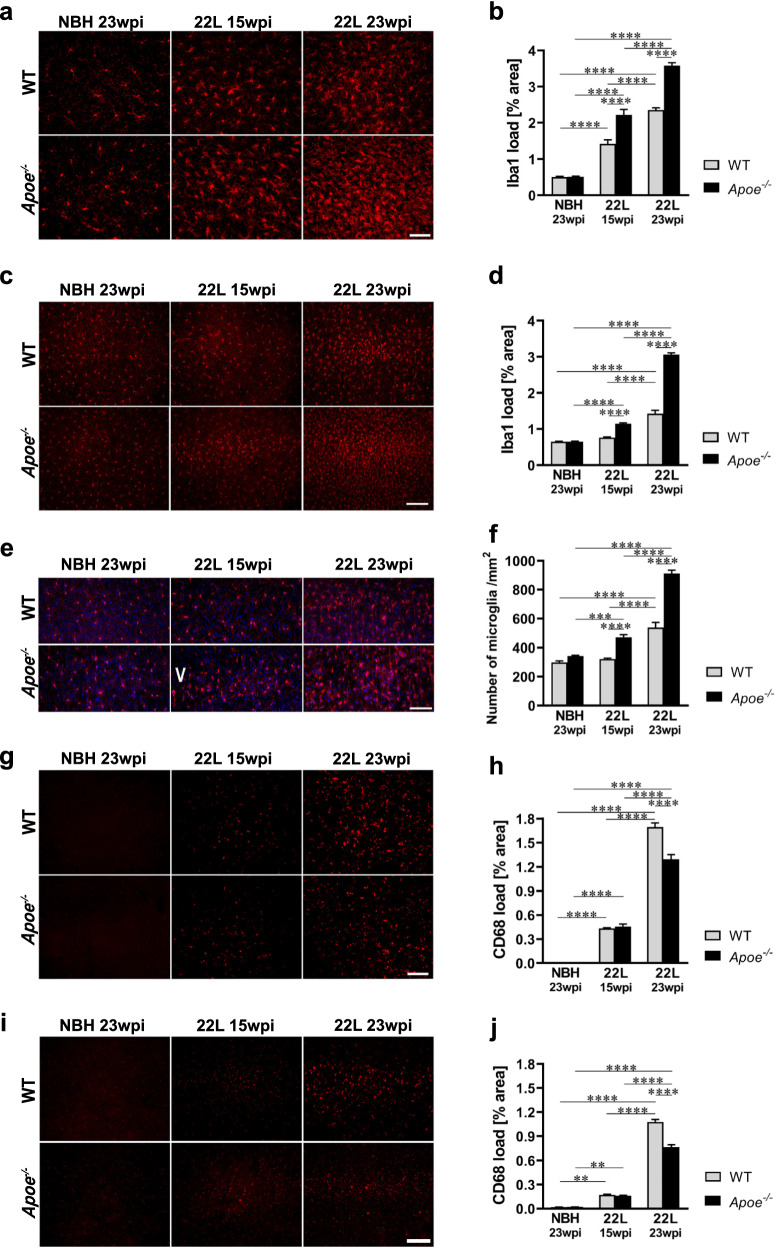
Exaggerated microglia activation in prion infected *Apoe*^*−/−*^ mice. **a** and **c** Representative microphotographs of coronal cross-sections through the VPN thalamic nucleus and the S1 primary somatosensory cortex immunostained against Iba1 from indicated animal groups, respectively. **b** and **d** Quantitative analysis of Iba1^+^ load in the VPN and the S1 cortex, respectively (n = 8–12 mice/group). **e** Representative microphotographs of Iba1/DAPI double stained microglia in the layer V of the S1 cortex. **f** Enumeration of Iba1^+^/DAPI^+^ microglia in the layer V of the S1 cortex (n = 6–8 mice/group). **g** and **i** Representative microphotographs of coronal cross-sections through the VPN and the S1 cortex immunostained against CD68 from indicated animal groups, respectively. **h** and **j** Quantitative analysis of CD68^+^ load in the VPN and the S1 cortex, respectively (n = 8–12 mice/group). While the Iba1^+^ load and the number of microglia increases earlier in the course of prion infection and is more robust in 22L *Apoe*^*−/−*^ mice compared to 22L WT mice, the CD68^+^ load is comparable between the genotypes at 15 wpi, while at 23 wpi it is lower in 22L *Apoe*^*−/−*^ mice compared to 22L WT mice. **b**, **d**, **f**, **h, j** *p* < 0.0001 (ANOVA); **p < 0.01, ***p < 0.001, and ****p < 0.0001 (Holm’s-Sidak’s post hoc test). All numerical values represent mean + SEM. Scale bars: 50 μm in **a**, **e**, and **g**, and 100 μm in **c** and **i**

Parallel to astrogliosis we analyzed activation of microglia at 15 wpi and 23 wpi. We quantified the load of Iba1^+^ and CD68^+^ cells in the VPN and in the S1 cortex as general and phagocytic microglia activation markers, respectively [57]. Similarly, to the extent of GFAP^+^ astrocytosis, at 15 wpi both 22L WT and 22L *Apoe*^−/−^ mice show significantly increased Iba1^+^ load in the VPN compared to their genotype-matched NBH controls (Fig. 6a, b) but only 22L *Apoe*^−/−^ mice show significantly elevated Iba1^+^ load in the S1 cortex (Fig. 6c, d). At 23 wpi both 22L WT and 22L *Apoe*^−/−^ mice show significantly higher Iba1^+^ load in the VPN and the S1 cortex over their NBH controls (Fig. 6a–d; Additional file 1: Fig. S6). At either time point, the Iba1^+^ load in 22L *Apoe*^−/−^ mice is higher relative to that in 22L WT mice. At 15 wpi it is 1.6-fold higher in the VPN (*p* < 0.0001) and 1.5-fold higher in the S1 cortex (*p* < 0.0001), while at 23 wpi 1.5-fold higher in the VPN (*p* < 0.0001) and 2.1-fold higher in the S1 cortex (*p* < 0.0001). In addition to the Iba1^+^ load, we enumerated density of Iba1^+^ microglial cells in the layer V of the S1 cortex, which shows the greatest microglia proliferation, consistently with the patterns of PrP deposition (Additional file 1: Fig. S6). In 22L *Apoe*^−/−^ mice the numerical density of microglia in the layer V is 1.5-fold higher at 15 wpi (*p* < 0.0001) and 1.7-fold higher at 23 wpi (*p* < 0.0001) relative to those in 22L WT mice, respectively (Fig. 6e, f).

CD68^+^ load shows significant increase in the VPN and in the S1 cortex already at 15 wpi both in 22L WT and 22L *Apoe*^−/−^ mice compared to their genotype-matched NBH controls (Fig. 6g–j). However, unlike Iba1^+^ load, there is no significant difference in the numerical values of the CD68^+^ load between 22L WT and 22L *Apoe*^−/−^ mice at 15 wpi. At 23 wpi the CD68^+^ load in both groups increases further, but in 22L *Apoe*^−/−^ mice represents 0.8-fold (*p* < 0.0001) and 0.7-fold (*p* < 0.0001) of those in 22L WT mice in the VPN and in the S1 cortex, respectively. These observations suggest considerably lower phagocytic activity of microglia in 22L *Apoe*^−/−^ mice relative to their overall activation compared to 22L WT mice. There is no significant difference in the Iba1^+^ load, numerical density of Iba1^+^ microglia, and CD68^+^ load in the VPN and in the S1 cortex between NBH inoculated WT and *Apoe*^−/−^ mice (Fig. 6a–j; Additional file 1: Fig S6).

**Fig. 7:**
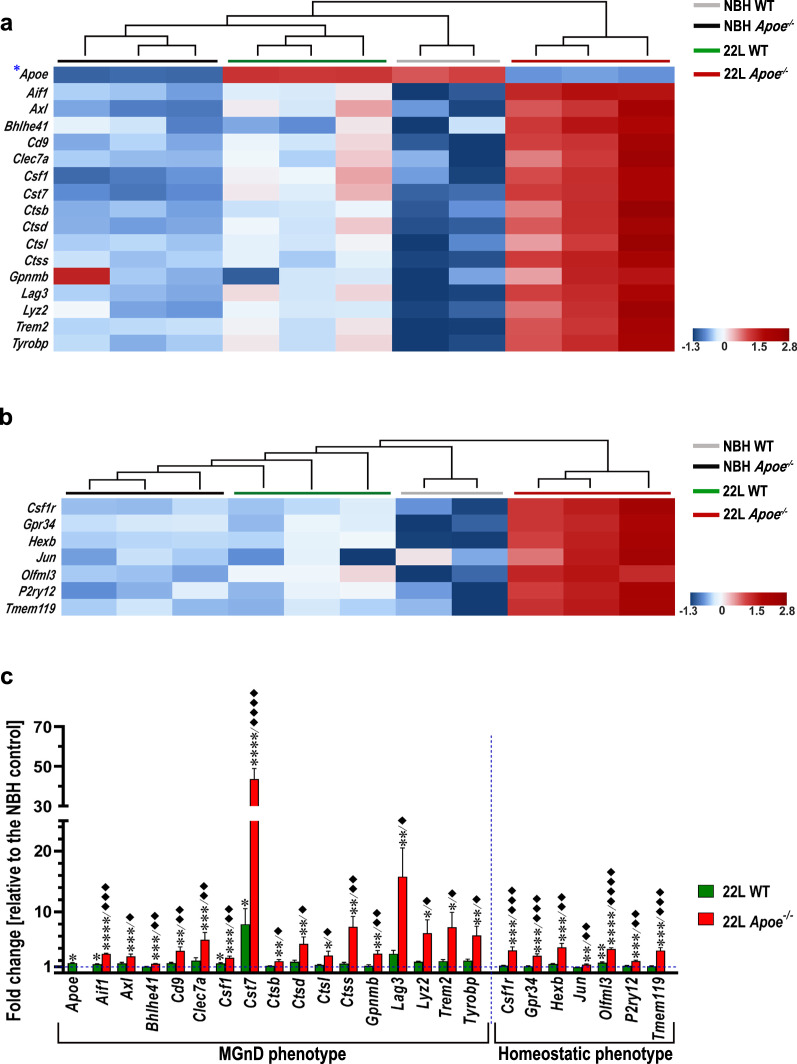
Prion infected *Apoe*^*−/−*^ mice feature marked upregulation of MGnD and M0 transcriptomic profiles. Shown are transcript heatmaps of nanoStringTM nCounter® expression data from NBH and 22L inoculated WT and *Apoe*^*−/−*^ mice, which were killed at 23 wpi. Microglia specific transcripts are split into **a** MGnD–specific, and **b** M0 (Homeostatic)—specific cassettes. Hierarchical cluster analysis shows salient separation of 22L *Apoe*^*−/−*^ mice from other experimental groups both for MGnD- and M0- specific cassettes, while 22L WT mice separate from NBH inoculated WT and *Apoe*^*−/−*^ mice within the MGnD-specific cassette but not within M0- specific cassette. **c** Fold change of nanoStringTM nCounter® values for MGnD and selected homeostatic (M0) microglia markers in 22L WT and 22L *Apoe*^−/−^ mice (n = 3 mice/group) relative to those in averaged NBH controls (n = 5 mice/group). **c** *p* < 0.05 to *p* < 0.0001 (ANOVA) analyzed for each gene separately; *p < 0.05 **p < 0.01 ***p < 0.001, and ****p < 0.0001 22L *Apoe*^−/−^ or 22L WT vs averaged NBH WT and NBH *Apoe*^−/−^; ^♦^*p* < 0.05, ^♦♦^*p* < 0.01, ^♦♦♦^*p* < 0.001, and ^♦♦♦♦^*p* < 0.0001 22L *Apoe*^−/−^ vs 22L WT (Holm’s-Sidak’s post hoc test following significant ANOVA). Data in **c** represent mean + SEM

Microglia specific transcripts split into MGnD—specific, and M0 (Homeostatic)—specific cassettes was characterized in animals killed at 23 wpi using the nanoStringTM nCounter® technology. Hierarchical cluster analysis of MGnD specific cassette shows clustering at individual group level (NBH WT, NBH *Apoe*^*−/−*^, 22L WT, and 22L *Apoe*^*−/−*^) and distinct separation of the 22L *Apoe*^*−/−*^ animals from other groups (Fig. 7a). Direct comparison of the nCounter® values reveals robust increase of MGnD markers in 22L *Apoe*^−/−^ mice relative to the averaged NBH controls: *Aif1* (3.1-fold*, p* < 0.0001)*, Axl* (2.7-fold*, p* < 0.001)*, Bhlhe41* (1.5-fold*, p* < 0.001)*, Cd9* (3.6-fold*, p* < 0.01)*, Clec7a* (5.5-fold*, p* < 0.001)*, Csf1* (2.5-fold*, p* < 0.001)*, Cst7* (43.6-fold*, p* < 0.0001), *Ctsb* (1.9-fold*, p* < 0.01)*, Ctsd* (4.8-fold*, p* < 0.01)*, Ctsl* (2.8-fold*, p* < 0.05)*, Ctss* (7.5-fold*, p* < 0.01)*, Gpnmb* (3.2-fold*, p* < 0.01)*, Lag3* (15.8-fold*, p* < 0.01)*, Lyz2* (6.5-fold*, p* < 0.05)*, Trem2* (7.5-fold*, p* < 0.05), and *Tyrobp* (6.2-fold*, p* < 0.01) (Fig. 7c)*.* In 22L WT mice the increase in some of MGnD markers also is observed but it is more modest compared to the degree of their upregulation in 22L *Apoe*^−/−^ mice. Statistically significant differences relative to the pooled NBH controls are noted for *Apoe* (1.6-fold*, p* < 0.05), *Aif1* (1.5-fold*, p* < 0.05)*, Csf1* (1.6-fold*, p* < 0.05)*,* and *Cst7* (8.0-fold*, p* < 0.05) (Fig. 7c). In direct comparison of the nCounter® values between 22L *Apoe*^−/−^ and 22L WT mice, there is a statistical post-hoc significance for all MGnD markers with the former group showing numerical advantage, except for *Apoe*, which is not expressed by 22L *Apoe*^−/−^ mice (Fig. 7c). Hierarchical cluster analysis of M0 specific cassette shows clustering within NBH WT and 22L *Apoe*^*−/−*^ groups with salient separation of the 22L *Apoe*^*−/−*^ animals from other experimental groups (Fig. 7b). Direct comparison of the nCounter® values shows significantly increased expression of several gene markers typical for M0 homeostatic microglia in 22L *Apoe*^−/−^ mice relative to the averaged NBH controls: *Csf1r* (3.7-fold*, p* < 0.001)*, Gpr34* (2.8-fold*, p* < 0.001)*, Hexb* (4.2-fold*, p* < 0.001)*, Jun* (1.4-fold*, p* < 0.01)*, Olfml3* (3.9-fold*, p* < 0.0001)*, P2ry12* (1.9-fold*, p* < 0.001), and *Tmem119* (3.7-fold*, p* < 0.001) (Fig. 7c). In contrast, in 22L WT mice only *Olfml3* expression is significantly increased (1.7-fold*, p* < 0.01) (Fig. 7c), however other M0 markers show no significant repression. Values in the parentheses both for 22L *Apoe*^−/−^ and 22L WT mice denote fold change and statistical significance relative to the averaged NBH controls. In direct comparison of the nCounter® values between 22L *Apoe*^−/−^ and 22L WT mice, there is a statistical post-hoc significance for all M0 markers with the former group showing numerical advantage (Fig. 7c). Significant upregulation of the *P2ry12* and *Tmem119* transcript in 22L *Apoe*^*−/−*^ mice at 23 wpi relative to NBH *Apoe*^*−/−*^ and 22L WT mice was independently confirmed by qRT-PCR (Additional file 1: Fig. S7a, b). Likewise the load of TMEM119^+^ microglia in the S1 cortex in 22L *Apoe*^*−/−*^ mice is 1.4-fold higher (*p* < 0.0001) than that in 22L WT mice at 23 wpi (Additional file 1: Fig. S7c, d). 22L infected mice of both genotypes feature significant increase in the TMEM119^+^ load compared to their genotype matched NBH controls. NBH *Apoe*^*−/−*^ mice also shows modestly higher TMEM119^+^ load compared to NBH WT mice.

### In prion infected mice lacking apoE activated microglia show impaired phagocytic activity

**Fig. 8:**
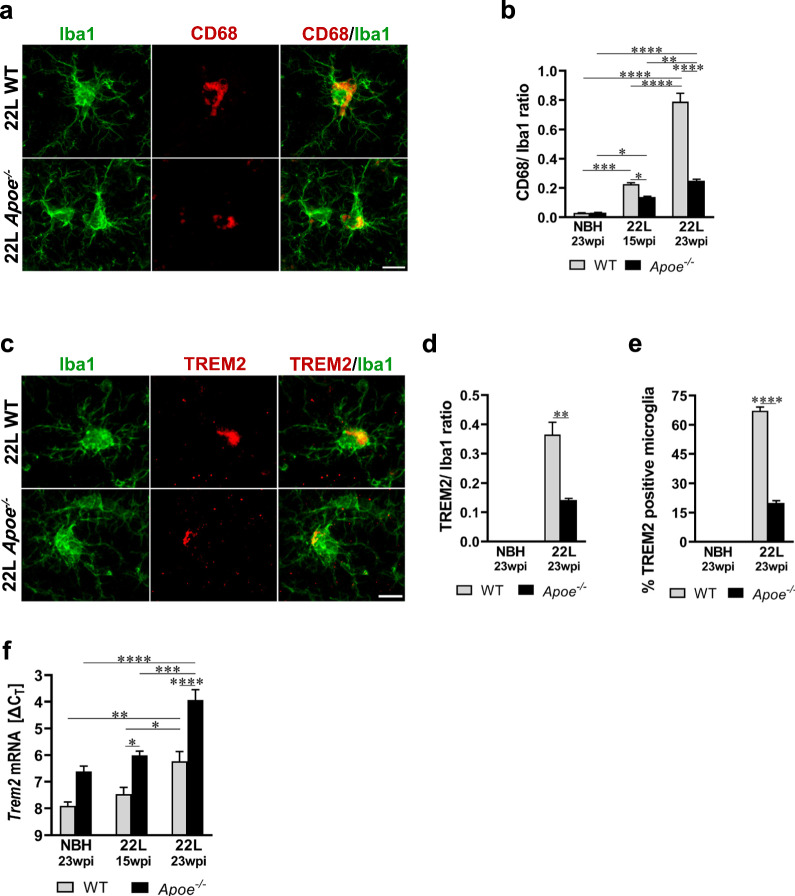
Attenuated microglial phagocytic in prion infected *Apoe*^*−/−*^ mice. **a** and **c** Representative LSCM images of CD68/Iba1 and TREM2/Iba1 double immunostained microglia in the S1 cortex of 22L inoculated WT and *Apoe*^*−/−*^ mice at 23 wpi, respectively. **b**, **d** Quantitative analysis of CD68/Iba1 and TREM2/Iba1 ratio in the S1 cortex, respectively; and **e** enumeration of % TREM2^+^ microglia in the layer V of the S1 cortex in indicated animal groups (n = 8–12 mice/group in **b** and n = 6–7 mice/group in **d** and **e**). **f** Analysis of *Trem2* mRNA level. The qRT-PCR results are presented as the ΔC_T_ values (n = 3–11 mice/group). Microglia in 22L *Apoe*^*−/−*^ mice feature attenuated expression of CD68 and TREM2 protein, which are microglial phagocytic activation markers compared to 22L WT mice, despite upregulation of *CD68* and *Trem2* transcript. **b** and **f** *p* < 0.0001 (ANOVA); *p < 0.05, **p < 0.01, ***p < 0.001, and ****p < 0.0001 (Holm’s-Sidak’s post hoc test). **d** and **e** **p < 0.01, and ****p < 0.0001 (two-tailed *t*-test with Welch’s correction). Values in **b**, **d**, **e**, and** f** represent mean + SEM. Scale bar: 10 μm in **a** and **c**

**Fig. 9:**
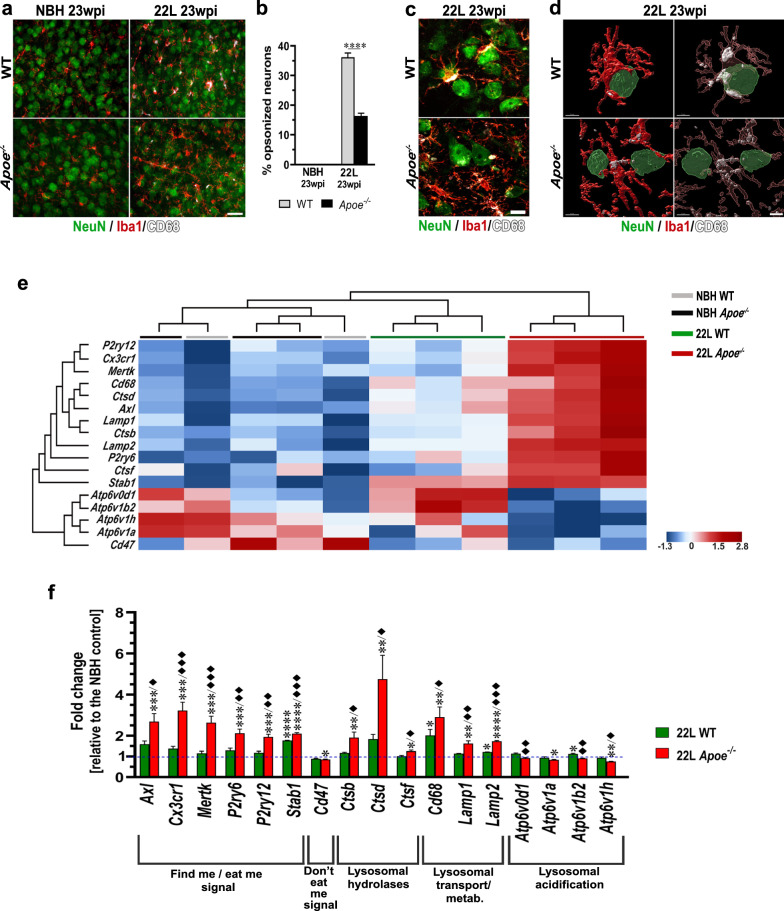
Impairment of microglia neuronophagy in prion infected *Apoe*^*−/−*^ mice**. a** Representative epifluorescent microphotographs of the S1 somatosensory cortex triple immunostained against NeuN neuronal marker and CD68 and Iba1 microglia markers in mice from indicated experimental groups at 23 wpi. **b** Enumeration of neurons opsonized by CD68^+^/Iba1^+^ microglia in the layer V of the S1 cortex (n = 6–7 mice/group). There is apparent reduction in the number of neurons coming in close contact with CD68^+^/Iba1^+^ microglia in 22L *Apoe*^*−/−*^ mice compared to 22L WT mice. **c** Representative LSCM images of NeuN/Iba1/CD68 triple immunostained sections and **d** 3-D reconstructions of LCSM stacks detailing neuronal opsonization by phagocytically activated (CD68^+^) microglia. In the right panel intracellular CD68 staining (white) is visualized by rendering Iba1 mask transparent. 3-D reconstructions of LCSM stacks details close enveloping of NeuN^+^ neurons by CD68^+^/Iba1^+^ microglia in in 22L WT mice and evidence for reduction in close neuronal/microglia contacts in 22L *Apoe*^*−/−*^ mice. **e** Shown are transcripts heatmaps of nanoStringTM nCounter® expression data for efferocytosis and endosomal/lysosomal pathways related genes in NBH and 22L inoculated WT and *Apoe*^*−/−*^ mice at 23 wpi. Individual 22L inoculated WT and *Apoe*^*−/−*^ mice but not NBH inoculated controls, cluster at group level. Clustering analysis shows clear separation of 22L *Apoe*^*−/−*^ animals from other groups. **f** Fold change of nanoStringTM nCounter® values for efferocytosis and phagocytosis markers in 22L WT and 22L *Apoe*^−/−^ mice (n = 3 mice/group) relative to those in averaged NBH controls (n = 5 mice/group). **b** ****p < 0.0001 (two-tailed *t*-test with Welch’s correction). **f** *p* < 0.05 to *p* < 0.0001 (ANOVA) analyzed for each gene separately; *p < 0.05 **p < 0.01 ***p < 0.001, and ****p < 0.0001 22L *Apoe*^−/−^ or 22L WT vs. NBH; ^♦^*p* < 0.05, ^♦♦^*p* < 0.01, and ^♦♦♦^*p* < 0.001 22L *Apoe*^−/−^ vs. 22L WT (Holm’s-Sidak’s post hoc test following significant ANOVA). Data in **b** and** f** represent mean + SEM. Scale bars: 30 μm in **a,** 10 μm in **c**, and 5 μm in **d**

We analyzed several aspects of microglial phagocytosis including quantification of the CD68 and TREM2 expression in the context of their Iba1 expression in the S1 cortex. 22L *Apoe*^−/−^ mice show significant reduction in the CD68:Iba1 ratio relative to 22L WT mice—0.6-fold change (*p* < 0.05) at 15 wpi and 0.3-fold change (*p* < 0.0001) at 23 wpi (Fig. 8a, b; Additional file 1: Fig. S8). The TREM2:Iba1 ratio also is reduced in 22L *Apoe*^−/−^ mice—0.4-fold change relative to 22L WT animals at 23 wpi (*p* < 0.01) (Fig. 8c, d; Additional file 1: Fig. S9). We also enumerated TREM2^+^ microglia in the layer V of the S1 cortex at 23 wpi. In 22L *Apoe*^−/−^ mice 20.0 ± 1.1% of microglia are TREM2^+^ while in 22L WT mice 67.2 ± 1.9% show immunodetectable TREM2 expression (3.4-fold difference, *p* < 0.0001) (Fig. 8e; Additional file 1: Fig. S9). Though there is reduction in the expression of CD68 and TREM2 protein by microglia, both *CD68* and *Trem2* transcripts are significantly upregulated in 22L *Apoe*^*−/−*^ mice relative to 22L WT animals (Figs. 7c, 8f, and 9f). Expression of CD68 in NBH inoculated mice is very low (CD68:Iba1 ratio ~ 0.03) and shows no significant differences between WT and *Apoe*^−/−^ animals (Fig. 8b, Additional file 1: Fig. S8), while expression of TREM2 is not detectable by immunohistochemistry in NBH inoculated mice of either genotype (Additional file 1: Fig. S9). In addition, we enumerated neurons, which are opsonized by phagocytically activated microglia, defined as being CD68^+^, and used this count as a surrogate marker of microglia neuronophagy. There is 2.2-times more neurons opsonized by microglia in 22L WT mice than in 22L *Apoe*^−/−^ mice in the layer V of the S1 cortex at 23 wpi (Fig. 9a, b) (*p* < 0.0001). Qualitative differences in the opsonization between the genotypes were further explored using LSCM imaging. It shows that in 22L WT mice opsonizing microglia tightly envelop neurons and feature high level of CD68 expression, while in 22L *Apoe*^−/−^ mice microglia/neuronal associations are loose while CD68 expression remains low (Fig. 9c, d). No close neuronal/microglia contacts are observed in NBH inoculated WT and *Apoe*^−/−^ controls (Fig. 9a, b).

Hierarchical cluster analysis of the nanoStringTM nCounter® gene expression data for the genes involved in the efferocytosis related signaling [28] and those involved in the endo/lysosomal pathway shows clear clustering within 22L WT and 22L *Apoe*^*−/−*^ groups and salient separation of 22L *Apoe*^*−/−*^ mice from all other animals (Fig. 9e). Direct analysis of the nCounter® values shows a significant upregulation in the group of *“find me/eat me”* signaling genes in 22L *Apoe*^−/−^ mice, which change relative to the averaged NBH controls is denoted along with the *p* value in the parentheses: *Axl* (2.7-fold*, p* < 0.001)*, Cx3cr1* (3.2-fold*, p* < 0.001)*, Mertk* (2.6-fold*, p* < 0.001)*, P2ry6* (2.1-fold*, p* < 0.001)*, P2ry12* (1.9-fold*, p* < 0.001), and* Stab1* (2.1-fold*, p* < 0.0001) (Fig. 9e, f). Inversely, expression of the *Cd47* gene relaying *“do not eat me”* signal is significantly repressed in 22L *Apoe*^−/−^ mice—0.85-fold change, relative to the NBH control (*p* < 0.05). In 22L WT mice expression of all *“find me/eat me”* signaling genes is modestly elevated but apart from *Stab1* (1.8-fold change relative to the NBH; *p* < 0.0001) it is not statistically significant (Fig. 9e, f). 22L WT mice also show reduction in *Cd47* expression (0.9-fold change relative to the NBH) which does not reach statistical significance. Differences in the expression of all *“find me/eat me”* genes between 22L *Apoe*^−/−^ and 22L WT groups are statistically significant with the former group showing greater degree of change (Fig. 9f). Expression of *Cd47* does not significantly differ between 22L inoculated WT and *Apoe*^−/−^ mice.

We also used the nanoStringTM nCounter® analysis to determine differences in the expression of genes involved in the endo/lysosomal pathway. In 22L *Apoe*^−/−^ mice there is a significant upregulation, denoted as a change relative to the averaged NBH controls, of lysosomal hydrolases *Ctsb* (1.9-fold*, p* < 0.01)*, Ctsd* (4.8-fold*, p* < 0.01)*,* and *Ctsf* (1.3-fold*, p* < 0.05) and lysosomal transport and metabolism markers *CD68* (2.9-fold*, p* < 0.01)*, Lamp1* (1.6-fold*, p* < 0.01)*,* and *Lamp2* (1.7-fold*, p* < 0.0001). In contrast expression of the genes encoding proteins responsible for lysosomal acidification (*Atp6v0d1*, *Atp6v1a*, *Atp6v1b2*, and *Atp6v1h*) is modestly reduced relative to the averaged NBH controls (0.76 – 0.92-fold change range) reaching statistical significance for *Atp6v1a* (*p* < 0.05) and *Atp6v1h* (*p* < 0.01) (Fig. 9e, f). In 22L WT mice lysosomal hydrolases and lysosomal transport and metabolism markers are only modestly upregulated relative to the NBH controls. The statistical significance is reached only for *CD68* (2.0-fold*, p* < 0.05) and *Lamp2* (1.2-fold change*, p* < 0.05). In direct comparison of the lysosomal hydrolases and the lysosomal transport and metabolism markers between 22L *Apoe*^−/−^ and 22L WT mice there is a statistical significance for all the genes with the former genotype showing greater degree of increase relative to the NBH controls (Fig. 9f). In contrast to 22L *Apoe*^−/−^ mice, the lysosomal acidification markers in 22L WT mice are either comparable to or higher than those in the averaged NBH controls (*Atp6v1b2;* 1.13-fold change*, p* < 0.05). In direct comparison between the 22L *Apoe*^−/−^ and WT groups, there is a statistical significance for all lysosomal acidification markers but *Atp6v1a* due to the opposite direction of change from the NBH control (Fig. 9f). This altered gene expression pattern indicates upregulation of neuronal/microglia signaling underlying neuronophagy or efferocytosis during prion pathogenesis, along with consistent increase in lysosomal hydrolases and lysosomal transport and metabolism markers with all changes enhanced in the absence of apoE. However, 22L *Apoe*^−/−^ mice also show oppositional reduction in the expression of lysosomal acidification markers, which suggests that absence of apoE renders lysosomal degradation inefficient.

### Absence of apoE aggravates inflammatory response associated with prion pathology

**Fig. 10:**
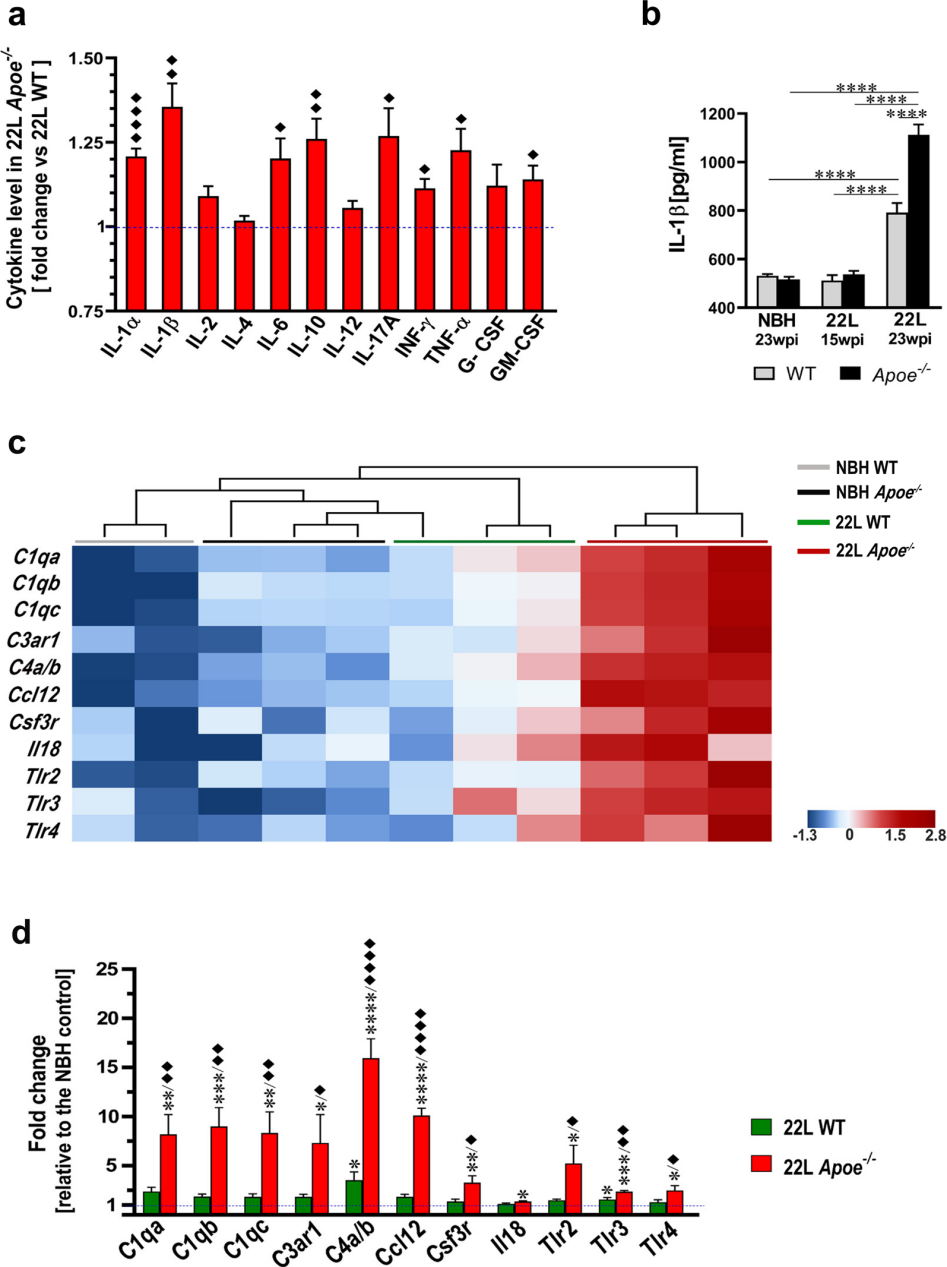
*Apoe*^*−/−*^ mice show aggravated inflammatory response to prion pathology.** a** Shown is quantitative analysis of multiple inflammatory cytokines in the brain cortex homogenate comparing 22L *Apoe*^−/−^ and 22L WT groups at 23 wpi. Values represent fold change in 22L *Apoe*^−/−^ mice relative to those in 22L WT mice (n = 5–7 mice/ group). **b** Quantitative analysis of IL-1β concertation in the brain cortex using ELISA in indicated animal groups (n = 5–8 mice/group). **c** Shown are transcripts heatmaps of nanoStringTM nCounter® expression data for chemokines and cytokine receptor genes from NBH and 22L inoculated WT and *Apoe*^*−/−*^ mice at 23 wpi. Clustering analysis shows strong separation of 22L *Apoe*^*−/−*^ mice from other groups. **d** Fold change of nanoStringTM nCounter® values for chemokines and cytokine receptors in 22L WT and 22L *Apoe*^−/−^ mice (n = 3 mice/group) relative to those in averaged NBH controls (n = 5 mice/group). **a** *p < 0.05, **p < 0.01, ****p < 0.0001 22L *Apoe*^−/−^ vs 22L WT (two-tailed *t*-test with Welch’s correction). **b** *p* < 0.0001 (ANOVA); ****p < 0.0001 (Holm’s-Sidak’s post hoc test). **d** *p* < 0.05 to *p* < 0.0001 (ANOVA) analyzed for each gene separately; *p < 0.05, **p < 0.01, ***p < 0.001, and ****p < 0.0001 22L *Apoe*^−/−^ or 22L WT vs NBH; ^♦^*p* < 0.05, ^♦♦^*p* < 0.01, and ^♦♦♦♦^*p* < 0.001 22L *Apoe*^−/−^ vs. 22L WT (Holm’s-Sidak’s post hoc test following significant ANOVA). Values in **a**, **b**, and **d** represent group mean + SEM

Increased brain level of inflammatory markers is an inherent feature of prion pathology. We used Mouse Inflammatory Cytokines Multi-Analyte ELISArray to compare the levels of inflammatory cytokines in the brain cortex homogenate between 22L WT and 22L *Apoe*^−/−^ mice killed at 23 wpi. Figure 10a depicts cytokine level in the brain cortex of 22L *Apoe*^−/−^ mice relative to those in 22L WT mice. There is significant increase in key pro-inflammatory cytokines IL-1α, IL-1β, IL-17A, IFN-γ, and TNF-α, which ranges from 1.1-fold to 1.4-fold (Fig. 10a). In addition, we detailed the level of IL-1β, which is a prime pro-inflammatory cytokine, in the brain cortex across all experimental groups (Fig. 10b). In mice killed at 15 wpi IL-1β level in 22L WT and 22L *Apoe*^−/−^ mice is comparable to those in genotype-matched NBH controls, while in mice killed at 23 wpi the IL-1β level is significantly increased both in 22L WT and 22L *Apoe*^−/−^ mice relative to the NBH controls (*p* < 0.0001). In 22L *Apoe*^−/−^ mice the IL-1β level is 1.4-fold higher relative to 22L WT mice (*p* < 0.0001).

Hierarchical cluster analysis of the nanoStringTM nCounter® gene expression data for the inflammatory response genes shows clear clustering of 22L *Apoe*^*−/−*^ animals and their salient separation from all other groups (Fig. 10c). Direct comparison of the nCounter® values between 22L *Apoe*^−/−^ mice and the averaged NBH controls shows a robust increase in the expression of genes encoding components of the C1 and the C4 complement complexes *C1qa* (8.2-fold*, p* < 0.01)*, C1qb* (9.0-fold*, p* < 0.001)*, C1qc* (8.3-fold*, p* < 0.01)*, C4a/b* (15.9-fold*, p* < 0.0001)*,* Complement Component 3a Receptor 1 (*C3ar1*) (7.3-fold*, p* < 0.05)*, Ccl12* (10.1-fold*, p* < 0.0001), Colony Stimulating Factor 3 Receptor (*Csf3r*) (3.2-fold*, p* < 0.01)*, Il18* (1.4-fold*, p* < 0.05), and Toll-like receptors *Tlr2* (5.2-fold*, p* < 0.05)*, Tlr3* (2.4-fold*, p* < 0.001)*, and Tlr4* (2.5-fold*, p* < 0.05) (Fig. 10d). In 22L WT mice upregulation of these genes is more modest and significant only for *C4a/b* (3.5-fold*, p* < 0.05), and* Tlr3* (1.6-fold*, p* < 0.05). The fold change and statistical significance in the parentheses denotes the difference relative to the averaged NBH controls. In direct comparison between 22L *Apoe*^−/−^ and 22L WT groups significant difference is noted for the expression of all the genes but *Il18* with the former group always showing greater degree of increase relative to the NBH controls (Fig. 10d).

### Prion induced neuronal loss and degeneration are aggravated by the lack of apoE

**Fig. 11:**
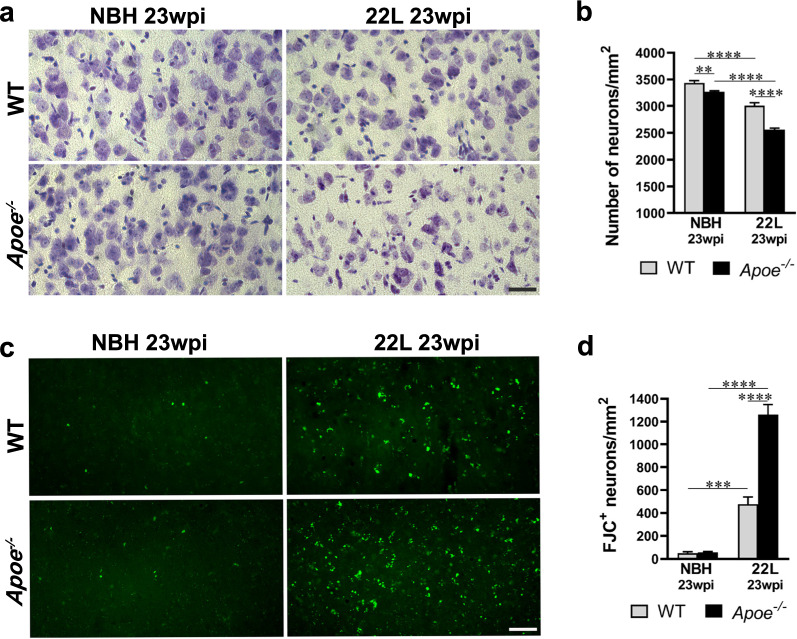
*Apoe*^*−/−*^ mice show aggravated neuronal loss and degeneration during prion infection.** a** and **c** Representative microphotographs of S1 cortex layer V neurons stained with cresyl violet and FJC from indicated animal groups, respectively. **b** and** d** Analysis of numerical density of cresyl violet and FJC stained neurons in the same area (n = 10–11 mice/group in **b** and n = 6–8 mice/group in **d**). 22L *Apoe*^*−/−*^ mice show greater neuronal loss and higher number of degenerating (FJC^+^) neurons compared to 22L WT mice. **b** and **d** *p* < 0.0001 (ANOVA); **p < 0.01, ***p < 0.001, and ****p < 0.0001 (Holm’s-Sidak’s post hoc test). Values in **b** and** d** represent mean + SEM. Scale bar: 30 μm in **a** and **c**

We enumerated pyramidal neurons in the layer V of the S1 cortex at 23 wpi and determined neuronal loss in the course of prion disease in 22L *Apoe*^−/−^ mice is nearly twice higher than that in 22L WT mice. In 22L *Apoe*^−/−^ mice there is 22% reduction in neuronal density (*p* < 0.0001), while in 22L WT mice the reduction is 13% (*p* < 0.0001) relative to their genotype-matched NBH control groups respectively (Fig. 11a, b). Of note, NBH inoculated *Apoe*^−/−^ mice have 5% reduced density of the layer V neurons compared to NBH inoculated WT mice (*p* < 0.01) (Fig. 11a, b). We also used staining with FJC, an anionic florescent dye, which selectively labels degenerating neurons [27, 67]. At 23 wpi 22L *Apoe*^−/−^ mice shows 2.6-times more FJC^+^ neurons in the layer V of the S1 cortex than 22L WT mice (*p* < 0.0001) which represents a higher rate of neuronal demise and degeneration in the former group (Fig. 11c, d). FJC staining of neurons in NBH *Apoe*^−/−^ and WT control groups was negligible.

## Discussion

Though prionoses share numerous aspects of pathogenesis with AD, where *APOE* genotype controls both the disease risk [20, 81] and the rate of symptoms progression [17, 21, 71, 75], the role of apoE in the pathomechanisms of prionoses remains unestablished. Our work shows that the total brain level of the apoE protein and that of *Apoe* transcript increases significantly in prion infected mice what is consistent with previous reports [13, 74]. We also found prion infection is associated with cell-type specific shift in the apoE production. Expression of apoE by activated astrocytes is downregulated, whereas apoE production by microglia is increased and thus responsible for the upregulation of the total brain apoE level what may have pathology-modulating consequences. There likely are structural and functional differences between astrocyte and microglia derived apoE particles. Whilst astrocyte secrete apoE in the form of HDL like particles, microglia feature limited expression of the ATP binding cassette subfamily A member 1, which transfers lipids upon apoE. Thus limited lipidation of microglia derived apoE particles has been postulated (reviewed in [19]). It also has been hypothesized that apoE secreted by astrocyte and that secreted by microglia may differentially regulate characteristics and function of activated microglia in neurodegeneration. Lipidated apoE may act as an opsonin facilitating efferocytosis of demising neurons and clearance of cellular remnants promoting phagocytic characteristics of microglia [61, 69, 80, 82]. Steep increase in the *Apoe* transcript level is an inherent feature of microglia activation and it was demonstrated in MGnD microglia isolated from brains of Tg mice representing AD and other neurodegenerative diseases [45]. MGnD microglia have been considered neurodegeneration promoting and the expression of apoE has been proposed to facilitate molecular signature of this phenotype through induction of specific transcription factors and the induction of the expression of miR-155 [9, 45]. Along these concepts both reduction of the apoE expression by reactive astrocytes and upregulation of the apoE expression in activated microglia we observed in 22L WT mice can be considered disease promoting.

To elucidate the net effect of apoE in the prion pathogenesis we prion inoculated *Apoe*^*−/−*^ mice. There is a single research study utilizing the *Apoe*^*−/−*^ mouse model, which was published 25 years ago by Tatzelt et al., and explored a hypothesis of apoE being a conformational chaperone of PrP^Sc^ and a catalyst of the PrP^C^ → PrP^Sc^ conversion [74]. The main quantitative readout of this study was the survival of *Apoe*^*−/−*^ mice intracerebrally inoculated with the RML strain, which was found indistinguishable from that of WT mice, and refuted the initial hypothesis explored by this study. Detailed prion pathology including astrocyte and microglia reaction was only superficially explored and was not presented in the form of quantitative metrics. We reexamined here the role of apoE in prion pathogenesis and contrary to this past report found that absence of apoE modestly, yet statistically significantly shortens incubation time of *Apoe*^*−/−*^ mice inoculated with the two distinct mouse adapted scrapie strains 22L and ME7. Unlike the work of Tatzelt et al. we did not inoculate the mice intracerebrally but selected the intraperitoneal inoculation route allowing for more protracted incubation time and the ascending involvement of the CNS, which we used to our advantage by examining prion related changes in the diencephalon and in the prosencephalon at different time points following the inoculation. Also instead of testing for behavioral metrics defying the end stage of prion disease (or survival) we focused on the earliest behavioral symptoms determining neuroinvasion. It is likely the combination of these three factors: prion strain selection, inoculation route, and use of early vs. endstage behavioral metrics, allowed us to demonstrate previously unappreciated effect of the *Apoe*^*−/−*^ on the accelerated occurrence of clinical symptoms of the disease. Though one cannot rule out the effect of *Apoe*^*−/−*^ on the efficiency of neuroinvasion, on the other hand there is no data to suggest an effect of *Apoe*^*−/−*^ on the PrP^C^ level outside the CNS. We also experimentally examined the effect of apoE on PrP^Sc^ replication and found no differences in the total PrP level and that of PrP^Sc^ between N2a/22L cells cultured in presence of natively lipidated apoE4 particles and those grown in control conditioned media from *Apoe*^*−/−*^ astrocytes. Quantification of prion pathology in *Apoe*^*−/−*^ mice showed significantly increased load of spongiform changes, modestly increased PrP^Sc^ level and marked upregulation of astro and microgliosis. Increased GFAP load was associated with upregulated expression of C3 protein and numerous transcriptomic markers typical of A1 neurotoxic astrocyte phenotype. The C3^+^ A1 neurotoxic astrocytes are induced by the cytokine combination consisting of TNFα, IL-1α, and C1qa, which is secreted by activated microglia [49]. This astrocytic phenotype contributes importantly to neurodegeneration and neuronal demise in various neurodegenerative diseases including prionoses [38, 48, 83]. More importantly, absence of apoE is directly associated with earlier and more pronounced activation of microglia, which display reduced phagocytic characteristics, upregulation of MGnD profile markers, and increased secretion of inflammatory cytokines. As the PrP^Sc^ spread follows the connectomal pattern, in the intraperitoneal prion inoculation model the thalamus is affected prior to the brain cortex owing it to the ascending PrP^Sc^ spread along the sensory spinal cord pathways. At 15 wpi, activation of microglia in 22L *Apoe*^*−/−*^ mice is detectable both in the thalamus and in the somatosensory cortex, while in the 22L WT mice only in the thalamus, where it is significantly lower than that in *Apoe*^*−/−*^ mice. Prion infected *Apoe*^*−/−*^ mice show both significantly increased Iba1 load and the number of microglial cells compared to 22L WT mice. Strikingly, microglia in 22L *Apoe*^*−/−*^ mice display attenuated phagocytic characteristics including reduced expression of CD68 and TREM2 proteins. They also have reduced number of neurons opsonized by activated, CD68^+^ microglia despite greater number of degenerating neurons revealed by FJC staining. Transcriptomic analysis using nanoStringTM nCounter® technology reveals upregulation of genes involved in “find me/eat me” signaling pathway and suppression of *Cd47* expression, which mediates “don’t eat me” signal in neuronal/microglia cross talk. This was observed both in 22L WT and *Apoe*^*−/−*^ mice, but it was more pronounced in the absence of apoE. Consistently, we find an upregulation of lysosomal hydrolase genes and lysosomal transport and metabolism markers. However, expression of genes expressing lysosomal acidification machinery is reduced in 22L *Apoe*^*−/−*^ mice but not in 22L WT mice indicating impaired lysosomal degradation in the absence of apoE. Interestingly, though *CD68* and *Trem2* transcript is increased in 22L *Apoe*^*−/−*^ mice, the expression of CD68 and TREM2 proteins by 22L *Apoe*^*−/−*^ microglia is significantly reduced as shown by direct microglia immunocytochemistry. Taken together these data indicate that aggravation of PrP^Sc^ mediated neurodegeneration in *Apoe*^*−/−*^ mice sends stronger signal prompting microglia to clean up degenerating neurons yet despite their hyperactivation, in the absence of apoE microglia fail to engage in effective phagocytosis and lysosomal degradation of neuronal remnants and PrP^Sc^. This finding supports an essential role of apoE in promoting phagocytic characteristics and function of activated microglia.

TREM2 plays an important role in microglia activation and phagocytosis. Dysfunctional variants of TREM2 has been identified as a major generic risk factor for sporadic AD [72]. Marked upregulation of TREM2 also is a feature of prion pathology as shown by our work consistently with previous studies [86]. Furthermore, highly increased CSF level of soluble TREM2 has been found in several human prionoses and proposed as a biomarker for these entities [26]. Soluble TREM2 has been recognized for its pro-inflammatory and microglia activation properties [85]. Effects of *Trem2*^*−/−*^ on prion pathology has been previously studied and it was found that TREM2 ablation attenuates prion-induced microglia activation, yet produces no effect on the actual survival time [86]. In this study we found apoE ablation upregulates *Trem2* transcript compared to WT mice infected with 22L strain, yet expression of TREM2 protein by microglia in *Apoe*^*−/−*^ mice is inversely down-regulated. A plausible explanation of this phenomenon may be that in the absence of lipidated apoE, which is a potent TREM2 binding target [82], surface expressed TREM2 is excessively cleaved, producing soluble TREM2, which has neuroinflammation enhancing effect.

The transcriptomic analysis also reveals upregulation of MGnD transcriptomic markers in prion infected mice. The MGnD has been associated with chronic neurodegenerative diseases and it is considered to be neurodegeneration promoting [61, 69]. It features an unique transcriptomic profile characterized by upregulation of genes related to phagocytosis, inflammation, and lipid metabolism including *Aif1, Apoe, Axl, Bhlhe41, Cd9, Clec7a, Csf1, Cst7*, *Ctsb, Ctsd, Ctsl, Ctss, Gpnmb, Lag3, Lyz2, Trem2*, and* Tyrobp* and the repression of the M0 (homeostatic) phenotype defining genes [9, 36, 45]. The MGnD can be induced in WT mice by injection of the apoptotic cells into the brain and their subsequent phagocytosis by homeostatic microglia [45]. MGnD induction and maintenance has been proposed to specifically depend upon expression of *Trem2* and *Apoe* and activation of the TREM2 signaling pathways [45, 69, 76]. Microglia in *Trem2*^*−/−*^ mice were shown not to transit to the MGnD, while lack of *Apoe* expression reduces neurodegeneration of the facial nerve nucleus following the VII nerve axotomy [36, 41, 45]. It has been proposed microglia expressed apoE upregulates several transcription factors and through these factors and miR-155 promotes the MGnD cassette specific transcript and represses the M0 phenotype defying genes [9, 45]. Consistent with this concept, microglia in 22L WT mice, show upregulation of MGnD specific transcript including *Trem2* and *Apoe*. In 22L *Apoe*^*−/−*^ mice lack of apoE does not prevent transition of homeostatic microglia to the MGnD, and even MGnD transcriptional markers reach significantly higher levels than those in 22L WT mice. However, this observation does not contradict the notion that in reactive microglia expression of apoE promotes the MGnD transcriptomic characteristics. ApoE still likely facilitates MGnD transition but lack of its expression can be overcome by high amount of neuronal debris derived from neurons degenerating en masse and in particular within a limited time frame.

Homeostatic microglia remain under the control of TGF-β signaling [8] and show a specific transcriptomic profile characterized by the expression of *Csf1r, Gpr34, Hexb, Jun, Olfml3, P2ry12, *and *Tmem119* [9, 36, 45]. Repression of the genes defining homeostatic microglia occurs during their transition to the MGnD and it is linked to the inverse upregulation of *Trem2* and *Apoe* transcripts as shown in elegant transcriptomic studies performed on primary microglia isolates [45]. In 22L *Apoe*^*−/−*^ mice microglia feature a significant upregulation rather than downregulation of *Csf1r, Gpr34, Hexb, Jun, Olfml3, P2ry12, *and *Tmem119* transcripts as shown by the nanoStringTM nCounter® analysis with the subsequent confirmation of upregulated *P2ry12 and Tmem119* transcript by qRT-PCR and TMEM119 protein by immunohistochemistry. These findings are consistent with the previously discussed notion that in MGnD apoE regulates several transcription factors and through these factors and miR-155 represses the homeostatic phenotype defying genes [9, 45]. We also found that in 22L WT mice expression of homeostatic microglia markers is not repressed but instead shows no significant differences when compared against NBH inoculated controls, with the exception of *Olfml3*, which is modestly increased. There are reports to indicate prion pathogenesis may be associated with disease-specific changes to homeostatic marker expression, which additionally may vary between stages of the disease. Hartmann et al. showed significant upregulation of anti-TMEM119 immunostaining in RML infected mice during the preclinical disease, while anti-P2RY12 immunostaining remained comparable to that of control animals [38]. Muth et al. showed that in fact anti-P2RY12 immunostaining in microglia becomes downregulated once the RML induced disease is allowed to progress [54]. However Carroll et al. who analyzed *Tmem119* and *P2ry12* transcripts at multiple time points in RML inoculated mice reported no consistent and significant changes. In CJD subjects, anti-TMEM119 immunostaining was found to be downregulated relatively to that in rapidly progressing AD, and CJD cases with concomitant Aβ pathology [46].

Upregulation of neuroinflammatory markers is typical of prion pathology [3]. Absence of apoE aggravates the neuroinflammatory cascade as shown by examining brain level of cytokines and expression of genes involved in innate immunity response including those for complement cascade proteins and toll-like receptors. Previous studies have suggested that in prion pathogenesis inflammatory cytokines exert their deleterious effect in concert rather than singularly drive the pathogenesis as transgenic mice lacking the expression of single cytokines show no change in the disease incubation period (reviewed in [3]). In contrast the absence of apoE results in upregulation of multiple cytokines, and leads to aggravation of prion related pathology.

**Fig. 12:**
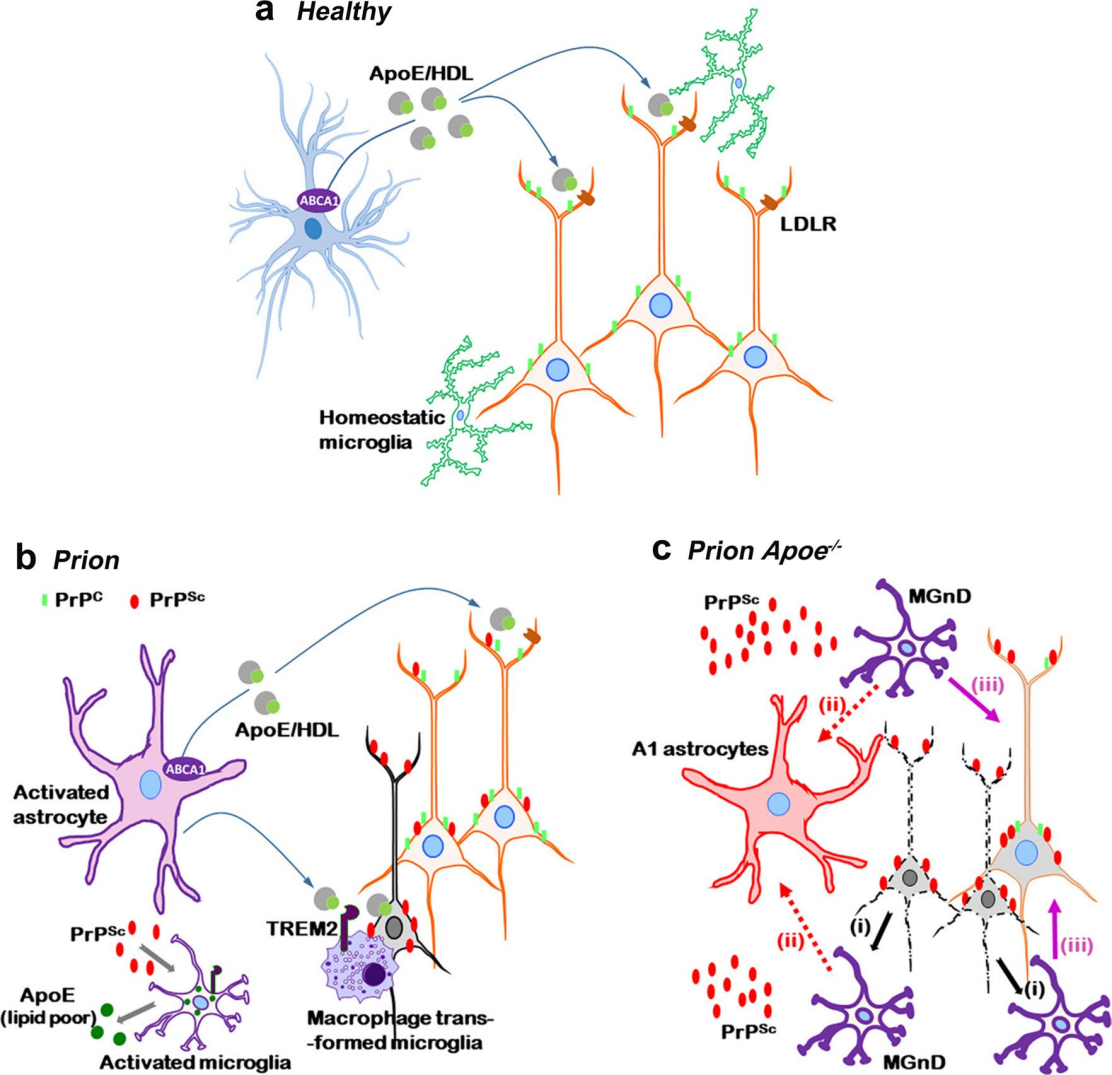
Schematic summary of hypotheses. **a** Under physiological conditions astrocytes secrete apoE/HDL particles, which are cleared by several receptors including the low-density lipoprotein receptor (LDLR). Homeostatic microglia surveil brain parenchyma and do not express apoE. **b** Replication of PrP^Sc^ within neurons is the initial culprit of prion pathogenesis and initiates neurodegeneration associated with early astrocyte and microglia activation. Activated microglia remove dying neurons and clear PrP^Sc^. ApoE/HDL secreted by astrocytes promote microglia phagocytosis by activating microglia TREM2 receptor pathway and by opsonization of degenerating neurons. Activated microglia also express lipid poor apoE, which more likely promotes their inflammatory phenotype rather than phagocytosis. **c** Absence of apoE renders clearance of PrP^Sc^ and removal of dying neurons by microglia ineffective. Neuronal remnants promote MGnD activation (i). MGnD microglia secrete a number of inflammatory cytokines including IL1α, TNF-α, and C1qa, which promote A1 neurotoxic astrocytes (ii) and are directly toxic to neurons (iii). Upregulated inflammatory cascade in turn promotes neuronal degeneration and increases the number of dying neurons, which removal is impaired. Thus global *Apoe*^*−/−*^ exacerbates prion pathology by propagating the vicious cycle of neuronal death and neuroinflammation. Our data also indicate homeostatic microglia transition to the MGnD even if they do not express apoE

The net contribution of microglia to prion pathogenesis is actively debated considering their opposing proinflammatory and phagocytic effects [3]. While clearance of PrP^Sc^ has been proposed to have a disease-limiting effect [12, 29, 30], microglia-driven neuroinflammation is deleterious to neurons [3, 10]. Conflicting outcomes of ablating microglia population in prion infected mice using a CSF1R inhibitor have been reported. While the initial study indicated attenuation of inflammatory markers and modestly prolonged survival [34], a more comprehensive recent study showed opposite results including shortening of the disease incubation period and increased PrP^Sc^ load supporting the view that phagocytic function of microglia in prionoses prevails over its proinflammatory effect [12]. We show here that in the absence of apoE prion microglia have diminished phagocytic capacity and enhanced pro-inflammatory characteristics resulting in dramatic aggravation of prion pathology including the higher number of degenerating neurons. Thus our work indicates the overall effect of apoE in prion pathogenesis is protective and contributes to the notion that microglia effectively attenuate the disease progression by timely phagocytosis of demising neurons and PrP^Sc^, which level in *Apoe*^*−/−*^ mice is increased (Fig. 12a, b). In the absence of apoE microglia fail to engage degenerating neurons, while neuronal remnants promote their MGnD characteristics. The MGnD microglia become toxic to neurons both directly through secretion of a number of inflammatory cytokines or indirectly through induction of A1 neurotoxic astrocytes. Upregulated inflammatory cascade in turn promotes neuronal degeneration and increases the number of dying neurons, which removal is impaired. Thus, we propose global *Apoe*^*−/−*^ exacerbates prion pathology by propagating the vicious cycle of neuronal death and neuroinflammation (Fig. 12c). Our findings further suggests that upregulation of astrocytic apoE expression and or its lipidation can be of therapeutic benefit in prion disease through enhancing phagocytic characteristics of activated microglia and promoting clearance of neuronal debris and PrP^Sc^. Likewise, selective suppression of the apoE expression in microglia may attenuate their acquisition of the MGnD characteristics and also ameliorate the pathogenesis through suppressing the neuroinflammatory response.

Clearance of PrP^Sc^ by microglia, has been proposed as a disease-limiting mechanism in prionoses, however details of engagement, engulfment, and disposal of PrP^Sc^ by microglia are unknown [12, 29, 30]. It also remains unexplored whether apoE is directly involved in the clearance of PrP^Sc^ by forming apoE/PrP^Sc^ complexes and facilitating their uptake by microglia as it is involved in AD pathogenesis by forming complexes with Aβ within the parenchymal plaques [77]. Further biochemical analyses focused on isolating apoE/PrP^Sc^ complexes from prion infected brains can shed light on this issue. Generation of Cre-Lox mice allowing for cell specific conditional *Apoe* knock out also can distinguish whether PrP^Sc^ interacting apoE is astrocyte or microglia derived. Though increased PrP^Sc^ level has been reported following ablating microglia population in prion infected mice using a CSF1R inhibitor [12] as well as in our study in *Apoe*^*−/−*^ mice, one needs to be however mindful that this effect may equally result from phagocytosis of PrP^Sc^ replicating neurons rather than from direct engagement of PrP^Sc^ deposited in the neuropil.

Unlike the *Apoe* gene in mouse*,* in humans, the *APOE* gene shows allelic diversity [55, 81]. The *APOE* genotype is recognized as the strongest determinant of risk for sporadic, late-onset AD. Possession of a single and double *ε4* allele increases AD risk ~ 3 and ~ 15-fold relative to *ε3/ε3* homozygotes, which is the most common *APOE* genotype, respectively [20, 55, 81]. The *APOE ε2* allele attenuates the disease risk but only in the absence of the *ε4* allele. Encoded by respective *APOE* alleles the apoE isoforms differentially affect Aβ clearance and deposition regulating AD risk [39, 51]. They also control inflammatory properties of activated microglia, which aggravate tau-mediated neuronal degeneration occurring downstream to Aβ deposition [70, 71]. Despite similarities between AD and prionoses the effect of *APOE* genotype in prion diseases has been only minimally explored. There is a study to suggest that *APOE ε4* carriers have nearly twofold increased risk of sporadic CJD [4]. However, the effect of apoE isoforms on the pathomechanism of prionoses has not been studied. Our findings, which implicate the effect of apoE in microglia mediated neurodegeneration justifies exploitation of the link between the *APOE* genotype and prion pathology. Given growing understanding of the regulatory role of apoE on microglia activation and characteristics in neurodegenerative disease and regulatory effect of human apoE isoforms on innate immune response [69, 71, 78] there is a strong premise to carry such studies in humans and in mice with targeted replacement of the murine *Apoe* gene for human *APOE* alleles [47].

## Conclusions

Our work demonstrates that absence of apoE impairs phagocytic clearance of neuronal remnants and PrP^Sc^ by microglia while aggravates microglia inflammatory response toward neurons, what has disease promoting consequences for the prion pathogenesis.

## Supplementary Information

**Additional file 1.Figure S1** Infection of Apoe-/- mice with ME7 mouse adapted scrapie strain causes significant shortening of the prion disease incubation period. **Figure S2** 22L infected Apoe-/- mice show faster progression of prion disease symptoms compared to 22L WT mice. **Figure S3** PrP deposition has predilection to the thalamus and to the layer V of the neocortex and is more prominent in 22L Apoe-/- mice. **Figure S4** ApoE does not alter total PrP or PrPSc level in N2A/22L cells. **Figure S5** Prion related expression of C3 by astrocytes is upregulated in the absence of apoE. **Figure S6** Activation of microglia in the course of prion pathogenesis shows predilection to the thalamus and to the layer V of the neocortex and is more prominent in 22L Apoe-/- mice. **Figure S7** Apoe-/- is associated with upregulation of P2RY12 and TMEM119 homeostatic microglia markers. **Figure S8** Expression of CD68 is reduced in Apoe-/- mice in the course of prion pathogenesis. **Figure S9** Microglia in 22L Apoe-/- mice have reduced TREM2 expression.

## Data Availability

Raw images and datasets that support the findings of this study are available from the corresponding author upon reasonable request.

## Abbreviations

Aβ: β-Amyloid
apoE: Apolipoprotein E
*Apoe*: ApoE gene (murine)
*APOE*: ApoE gene (human)
*APP*: Amyloid precursor protein gene (human)
B6: C57BL/6 mouse strain
CD68: Cluster of differentiation 68
CD230: Cluster of differentiation 230 (a.k.a PrP)
CSF: Cerebrospinal fluid
C3: Complement component 3
DAM: Disease associated microglia
DAPI: 4’, 6’-Diamidino-2-phenylindole
GFAP: Glial fibrillary acidic protein
HDL: High-density lipoprotein
Iba1: Ionized calcium binding adaptor molecule
LDLR: Low-density lipoprotein receptor
LSCM: Laser scanning confocal microscopy
M1: Primary motor cortex
MGnD: Microglial neurodegenerative phenotype
NBH: Normal brain homogenate
NeuN: Neuronal nuclear protein
PrP: Prion protein
PrP^Sc^: Scrapieform conformer of prion protein
S1: Primary somatosensory cortex
SEM: Standard error
TREM2: Triggering Receptor Expressed in Myeloid cells 2
wpi: Weeks post inoculation
VPN: Ventroposterior thalamic nucleus
WT: Wild type
