# Supplementary material for: Absence of Apolipoprotein E is associated with exacerbation of prion pathology and promotes microglial neurodegenerative phenotype
Source: Acta Neuropathol Commun. 2021 Sep 26;9:157. doi: 10.1186/s40478-021-01261-z (PMC8474943; doi:10.1186/s40478-021-01261-z)
Supplement: Supplementary file 1 — Additional file 1.Figure S1 Infection of Apoe-/- mice with ME7 mouse adapted scrapie strain causes significant shortening of the prion disease incubation period. Figure S2 22L infected Apoe-/- mice show faster progression of prion disease symptoms compared to 22L WT mice. Figure S3 PrP deposition has predilection to the thalamus and to the layer V of the neocortex and is more prominent in 22L Apoe-/- mice. Figure S4 ApoE does not alter total PrP or PrPSc level in N2A/22L cells. Figure S5 Prion related expression of C3 by astrocytes is upregulated in the absence of apoE. Figure S6 Activation of microglia in the course of prion pathogenesis shows predilection to the thalamus and to the layer V of the neocortex and is more prominent in 22L Apoe-/- mice. Figure S7 Apoe-/- is associated with upregulation of P2RY12 and TMEM119 homeostatic microglia markers. Figure S8 Expression of CD68 is reduced in Apoe-/- mice in the course of prion pathogenesis. Figure S9 Microglia in 22L Apoe-/- mice have reduced TREM2 expression. [file 40478_2021_1261_MOESM1_ESM.pdf]

Additional File 1: Supplementary information

Supplementary Figures and Supplementary Figure Legend

Absence of Apolipoprotein E Is Associated with Exacerbation of Prion Pathology and Promotes Microglial Neurodegenerative Phenotype

Joanna E. Pankiewicz[a, c, #], Anita M. Lizińczyk[a, #], Leor A. Franco[a],

Jenny R. Diaz[a], Mitchell Martá-Ariza[a], and Martin J. Sadowski[a-c]*

Departments of [a]Neurology, [b]Psychiatry, and [c]Biochemistry and Molecular Pharmacology,

New York University Grossman School of Medicine, New York, NY 10016

[#] These authors contributed equally.

Short title: *ApoE Modulates Prion Pathology*

*Correspondence: sadowm01@med.nyu.edu

Figure S1

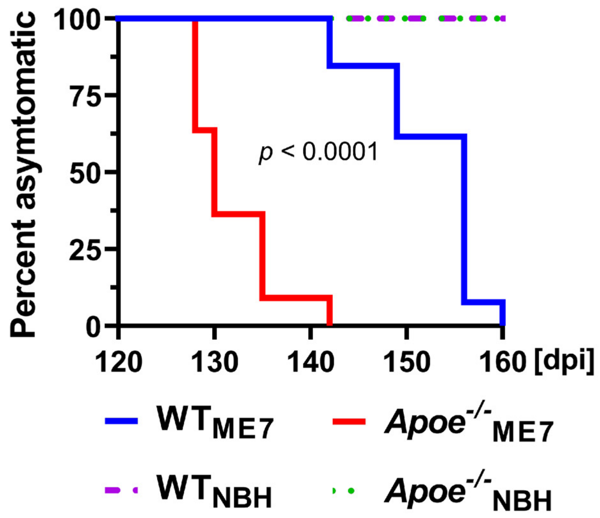
Percent asymtomatic
100
75
50
25
0
120
130
140
150
160
[dpi]
p < 0.0001
WT ME7
Apoe-/- ME7
WT NBH
Apoe-/- NBH

Fig. S1 Infection of *Apoe*$^{-/-}$ mice with ME7 mouse adapted scrapie strain causes significant shortening of the prion disease incubation period. To assure the effect of *Apoe*$^{-/-}$ on the prion pathology is not specific to the 22L strain we intraperitonealy inoculated 8 – 10 week-old WT and *Apoe*$^{-/-}$ mice of both sexes (~ 50%:50% female to male ratio) with ME7 infectious and normal brain homogenate (NBH). Unlike the 22L strain, the ME7 strain does not replicate in non-neuronal cells and has slightly longer incubation period. Shown is Kaplan-Meier estimator of the incubation time in ME7 and NBH inoculated WT and *Apoe*$^{-/-}$ mice. The x-axis denotes days post inoculation (dpi) while the y-axis a percent of animals, which remain asymptomatic from the initial groups of 11 - 13 ME7 and 22 - 23 NBH inoculated WT and *Apoe*$^{-/-}$ mice. $p < 0.0001$ denotes the significance between 22L WT and 22L *Apoe*$^{-/-}$ groups (Log-rank test). Differences between 22L *Apoe*$^{-/-}$ and NBH *Apoe*$^{-/-}$ and between 22L WT and NBH WT, which are not shown on the graph also are significant at $p < 0.0001$. The difference between NBH WT and NBH *Apoe*$^{-/-}$ is not statistically significant.

Figure S2

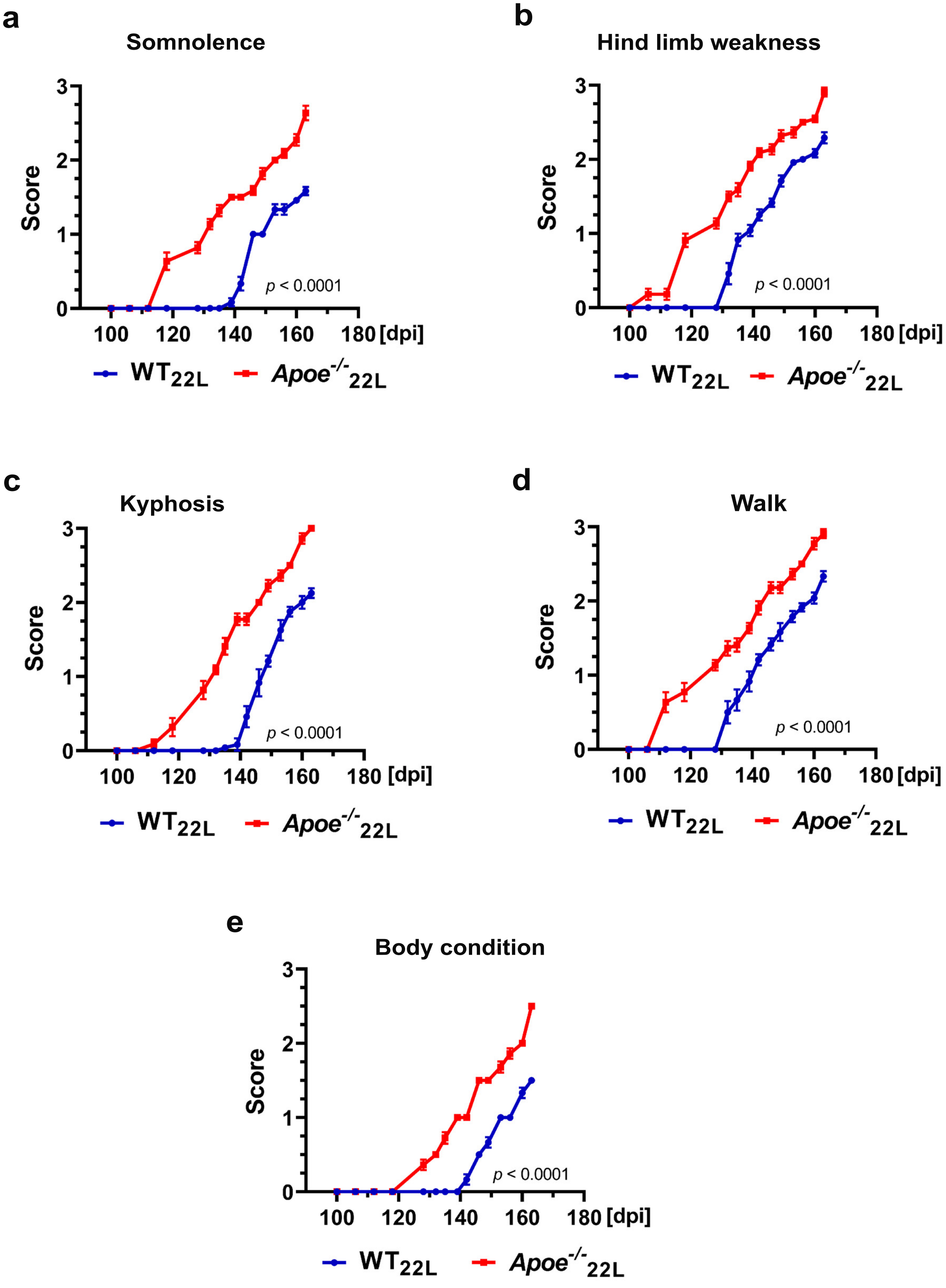
a
Somnolence
Score
p < 0.0001
[dpi]
WT22L
Apoe-/-22L
b
Hind limb weakness
Score
p < 0.0001
[dpi]
WT22L
Apoe-/-22L
c
Kyphosis
Score
p < 0.0001
[dpi]
WT22L
Apoe-/-22L
d
Walk
Score
p < 0.0001
[dpi]
WT22L
Apoe-/-22L
e
Body condition
Score
p < 0.0001
[dpi]
WT22L
Apoe-/-22L

Fig. S2 22L infected *Apoe*$^{-/-}$ mice show faster progression of prion disease symptoms compared to 22L WT mice. Shown are scores for **a** Somnolence, **b** Hind limb weakness, **c** Kyphosis, **d** Walk, and **e** Body condition, which were assigned based on the following criteria 0 = normal, 1 = subtle, 1.5 = mild, 2 = moderate, 2.5= advanced, and 3 = severe. The tally of all subscores makes the Total Scrapie Score depicted in Fig. 2b. The mice were serially assessed starting from the 100th day post inoculation by two independent examiners blinded to the animal genotype. All data represent mean ± SEM from n = 11 - 12 mice per group. **a** - **e** $p < 0.0001$ (2-way ANOVA).

Figure S3

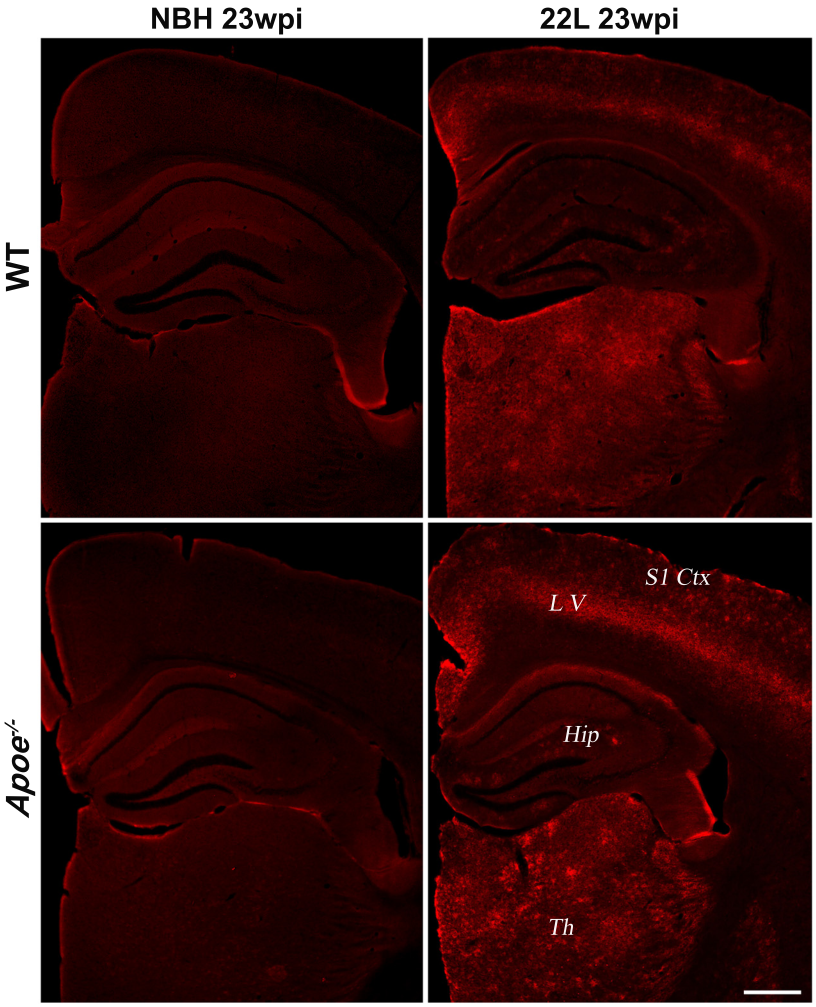

Fig. S3 PrP deposition has predilection to the thalamus and to the layer V of the neocortex and is more prominent in 22L *Apoe*$^{-/-}$ mice. Shown are representative microphotographs of coronal brain section from NBH and 22L inoculated WT and *Apoe*$^{-/-}$ mice at 23 wpi. The sections were immunostained against PrP. Scale bar: 350 µm. Abbreviations: Hip – hippocampus, L V – layer V, S1 Ctx – primary somatosensory cortex, and Th – thalamus.

Figure S4

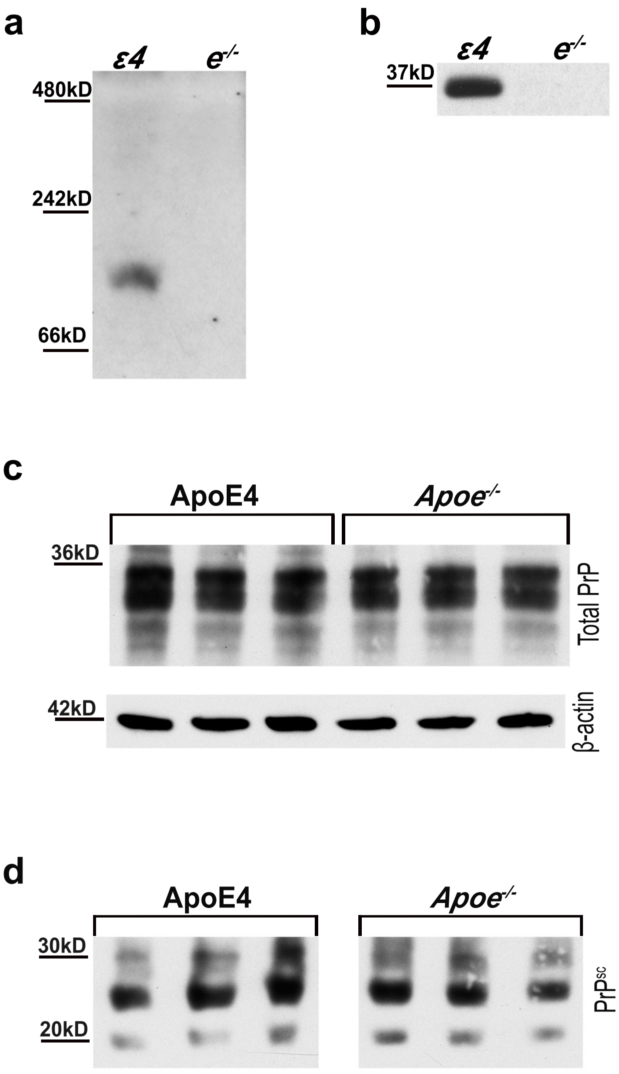
a
ε4
e-/-
480kD
242kD
66kD
b
ε4
e-/-
37kD
c
ApoE4
Apoe-/-
36kD
Total PrP
42kD
β-actin
d
ApoE4
Apoe-/-
30kD
20kD
PrPsc

Fig. S4 ApoE does not alter total PrP or PrP^{Sc} level in N2A/22L cells. **a** and **b** Shown are samples of conditioned media from immortalized astrocytes expressing human apoE4 ($\varepsilon 4$) and those from *Apoe*$^{-/-}$ astrocytes ($e^{-/-}$), which were resolved using native-PAGE and SDS-PAGE, respectively. **c** and **d** Show is immunoblot analysis of the total PrP protein and that of proteinase K (PK) resistant PrP^{Sc} in N2A/22L cells treated with astrocytic media containing natively lipidated apoE4 or control media from *Apoe*$^{-/-}$ astrocytes for 96 hrs., respectively. Also included is β-actin as the loading control in **c**.

Figure S5

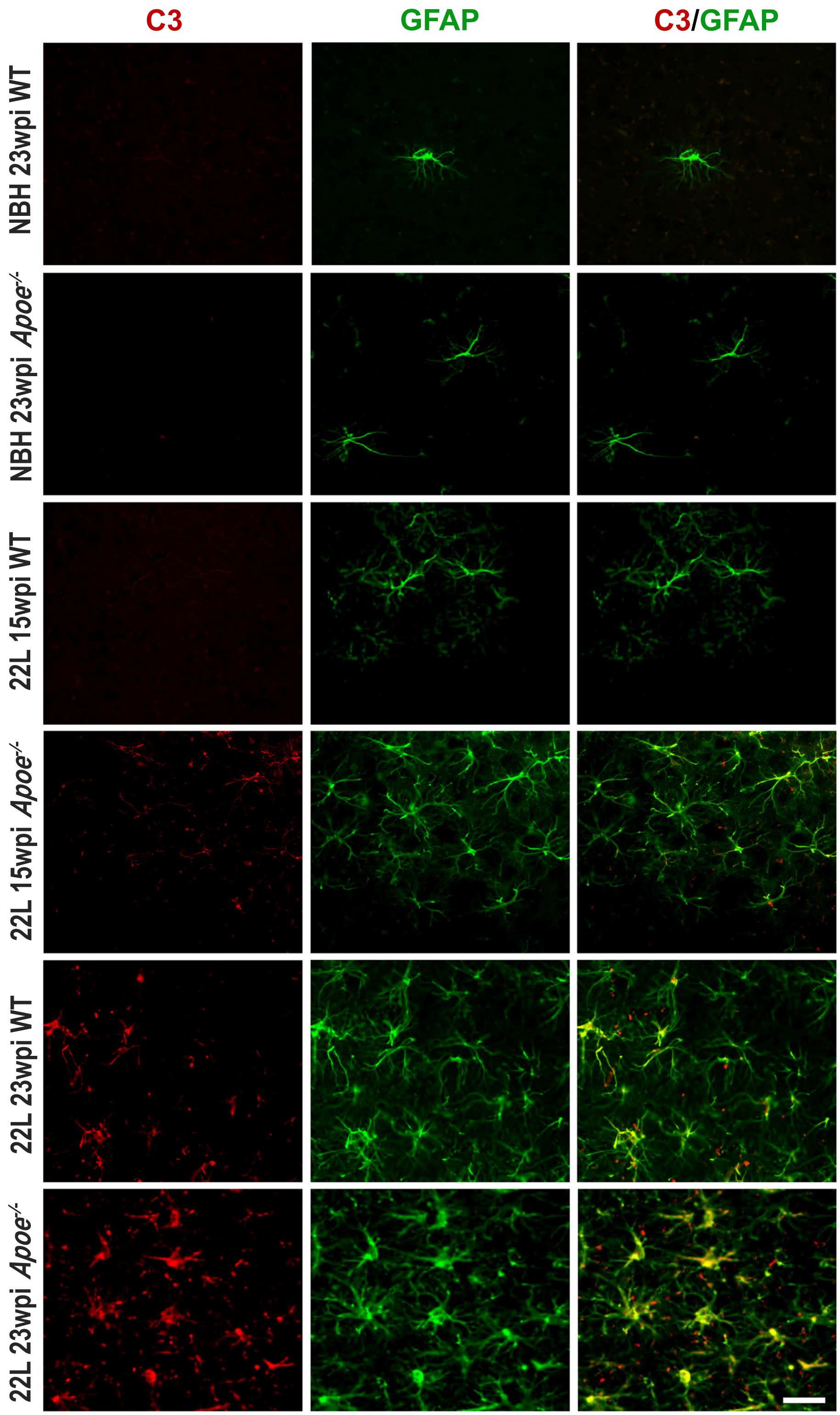
C3
GFAP
C3/GFAP
NBH 23wpi WT
NBH 23wpi *Apoe*-/-
22L 15wpi WT
22L 15wpi *Apoe*-/-
22L 23wpi WT
22L 23wpi *Apoe*-/-

Fig. S5 Prion related expression of C3 by astrocytes is upregulated in the absence of apoE. Shown are representative epifluorescent microphotographs of astrocytes in the layer V of the S1 cortex from mice of indicated experimental groups, which were double immunostained against C3 and GFAP. There is no C3 expression in astrocytes from control, NBH inoculated WT and *Apoe*$^{-/-}$ mice. At 15 wpi, C3 expression is detectable in astrocytes of 22L *Apoe*$^{-/-}$ mice but not in 22L WT mice, while at 23 wpi it is detectable in both genotypes but is significantly higher in 22L *Apoe*$^{-/-}$ mice. Scale bar: 40 µm.

Figure S6

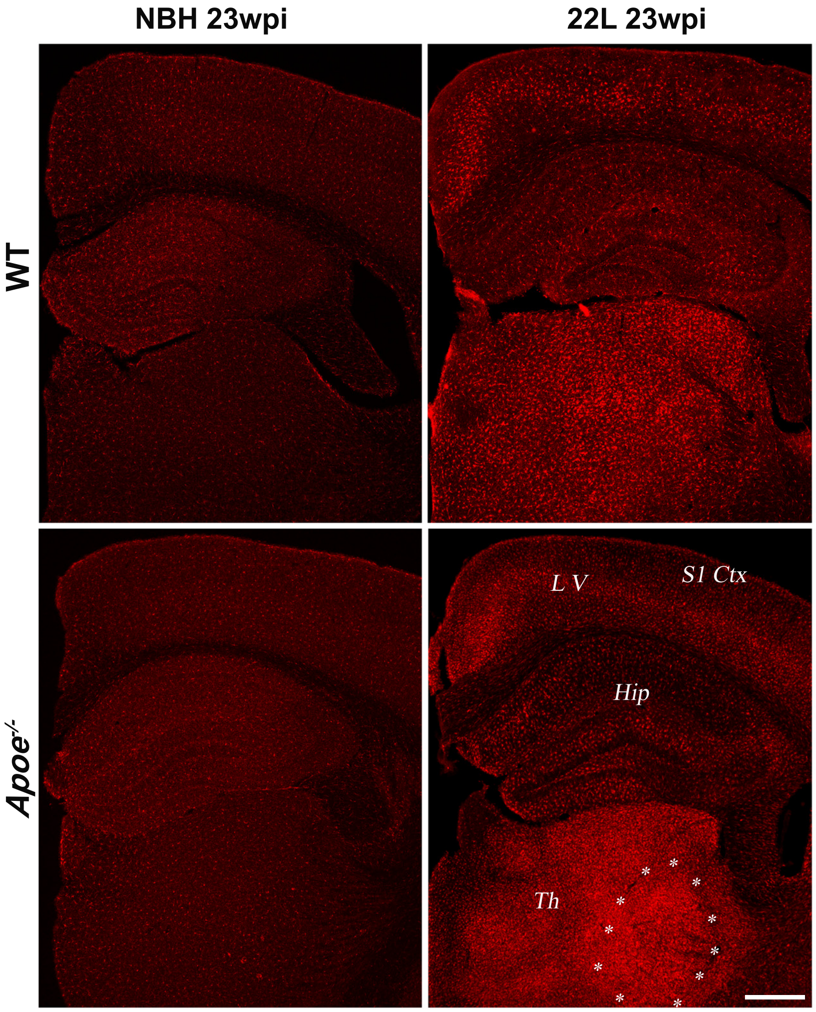

Fig. S6 Activation of microglia in the course of prion pathogenesis shows predilection to the thalamus and to the layer V of the neocortex and is more prominent in 22L *Apoe*$^{-/-}$ mice. Shown are representative microphotographs of coronal brain section from NBH and 22L inoculated WT and *Apoe*$^{-/-}$ mice at 23 wpi. The sections were immunostained against Iba1. Scale bar: 350 μm. Abbreviations: Hip – hippocampus, L V – layer V, S1 Ctx – primary somatosensory cortex, and Th – thalamus. Asterisks demarcates limits of the ventroposterior thalamic nucleus (VPN), which along with the S1 cortex was selected for the quantitative analysis of Iba^{+} microglia load.

Figure S7

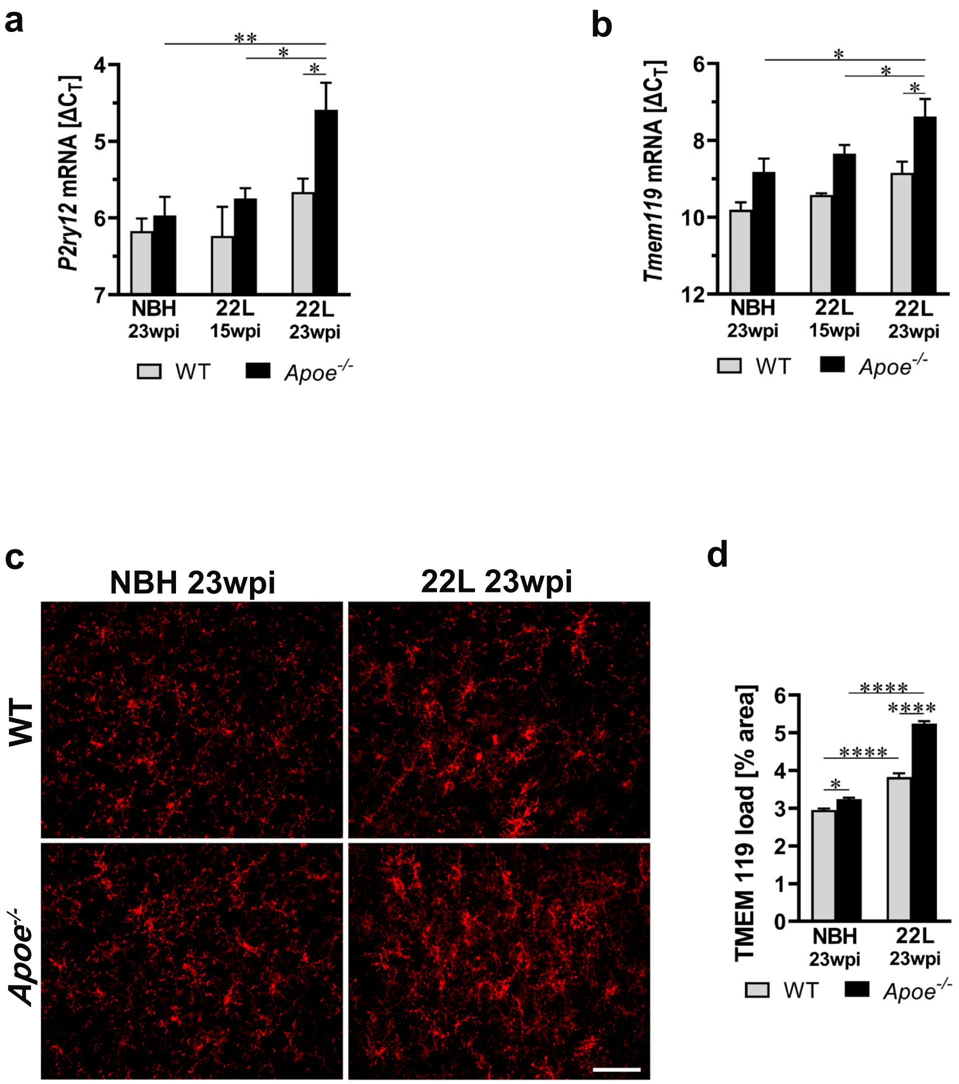
a
**
*
*
4
5
6
7
P2ry12 mRNA [ΔC_T]
NBH 23wpi
22L 15wpi
22L 23wpi
WT
Apoe-/-
b
*
*
*
6
8
10
12
Tmem119 mRNA [ΔC_T]
NBH 23wpi
22L 15wpi
22L 23wpi
WT
Apoe-/-
c
NBH 23wpi
22L 23wpi
WT
Apoe-/-
d
****
****
****
*
0
1
2
3
4
5
6
TMEM 119 load [% area]
NBH 23wpi
22L 23wpi
WT
Apoe-/-

Fig. S7 *Apoe*$^{-/-}$ is associated with upregulation of P2RY12 and TMEM119 homeostatic microglia markers. Analysis of **a** *P2ry12* and **b** *Tmem119* mRNA level. The qRT-PCR results are presented as the ΔC_T values (n = 3 - 11 mice/group). **c** Shown are representative epifluorescent microphotographs of microglia in the layer V of the S1 cortex from mice of indicated experimental groups, which were immunostained against TMEM119 and **d** the quantitative analysis of TMEM119 load in the S1 cortex, respectively (n = 5 - 7 mice/group). **a**, **b**, and **d** $p < 0.0001$ (ANOVA); *$p < 0.05$, **$p < 0.01$, and ****$p < 0.0001$ (Holm's-Sidak's post hoc test). Values in **a**, **b**, and **d** represent mean + SEM. Scale bar: 40 μm in **c**.

Figure S8

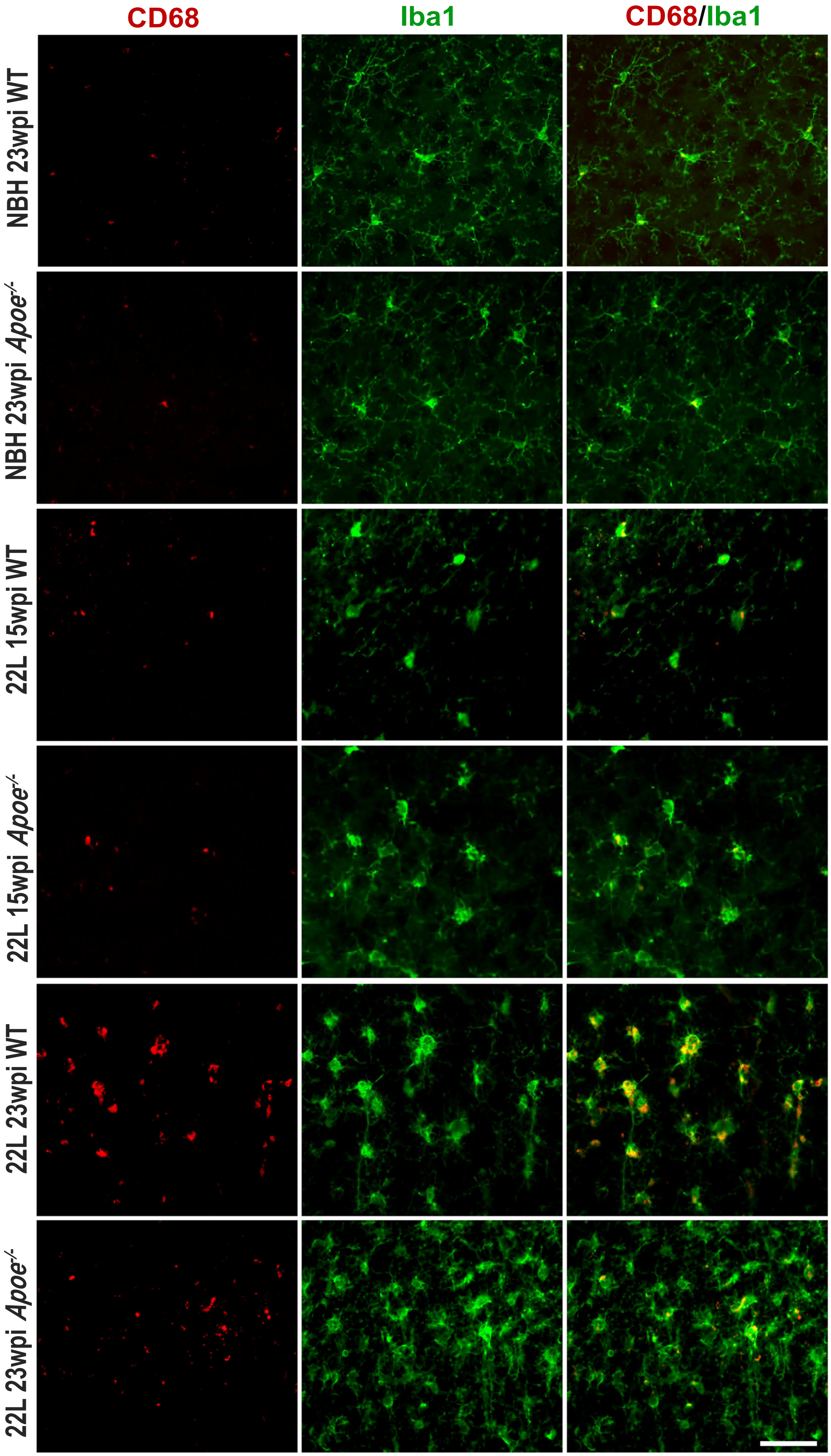
CD68
Iba1
CD68/Iba1
NBH 23wpi WT
NBH 23wpi *Apoe*-/-
22L 15wpi WT
22L 15wpi *Apoe*-/-
22L 23wpi WT
22L 23wpi *Apoe*-/-

Fig. S8 Expression of CD68 is reduced in *Apoe*$^{-/-}$ mice in the course of prion pathogenesis. Shown are representative epifluorescent microphotographs of microglia in the layer V of the S1 cortex from mice of indicated experimental groups, which were double immunostained against CD68 and Iba1. There is minimal CD68 expression by microglia in the control NBH inoculated WT and *Apoe*$^{-/-}$ mice. At 15 wpi, CD68 expression in 22L animals is modestly increased comparable to the controls and it is similar between 22L WT and 22L *Apoe*$^{-/-}$ mice despite, much greater microglia activation in the latter group. At 23 wpi, 22L WT mice show much higher CD68 expression than 22L *Apoe*$^{-/-}$ mice despite inverse degree of microglia activation revealed by anti-Iba1 immunostaining. Scale bar: 40 μm.

Figure S9

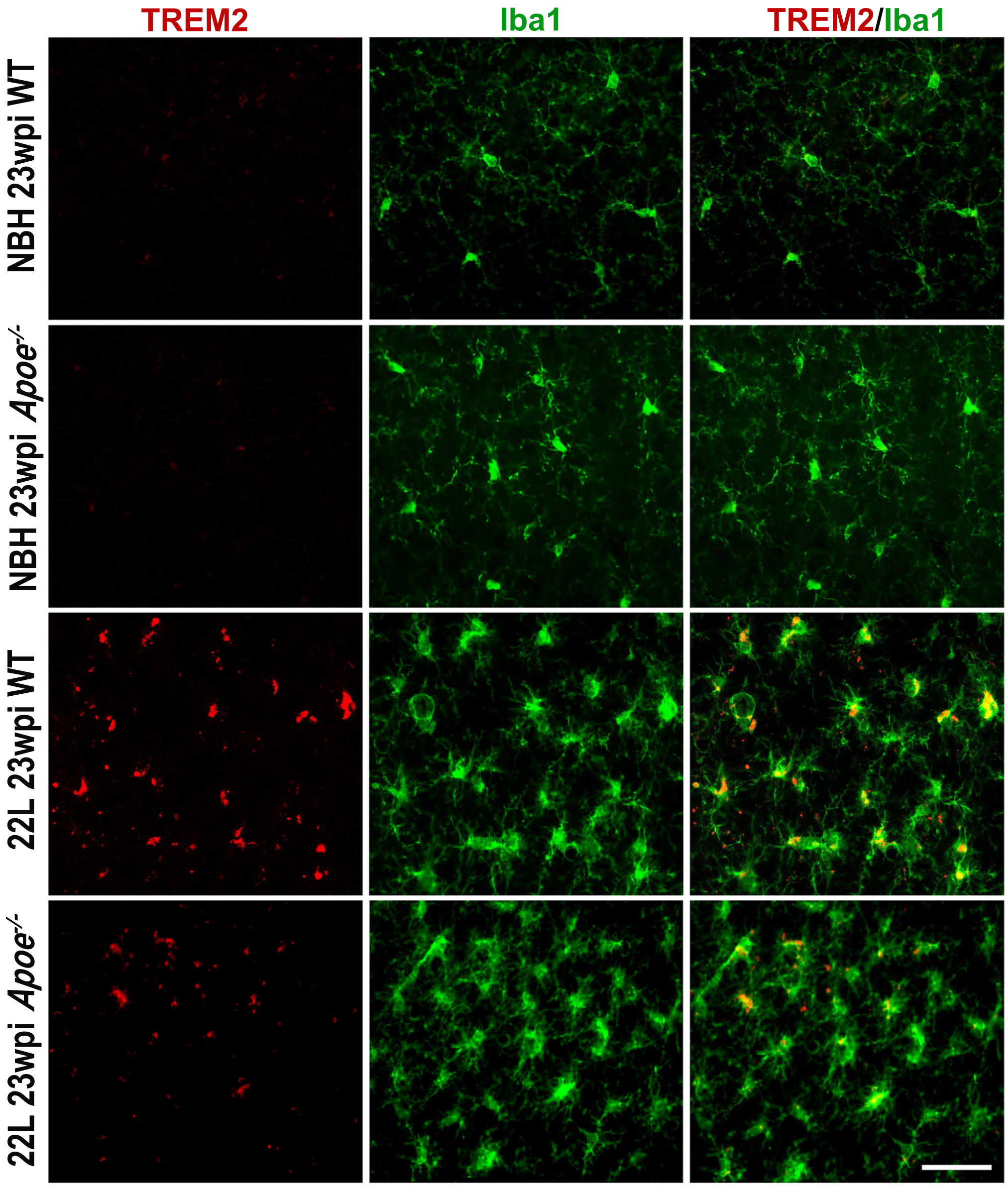
TREM2
Iba1
TREM2/Iba1
NBH 23wpi WT
NBH 23wpi *Apoe*$^{-/-}$
22L 23wpi WT
22L 23wpi *Apoe*$^{-/-}$

Fig. S9 Microglia in 22L *Apoe*$^{-/-}$ mice have reduced TREM2 expression. Shown are representative epifluorescent microphotographs of microglia in the layer V of the S1 cortex from mice of indicated experimental groups, which were double immunostained against TREM2 and Iba1. While there is no TREM2 expression in microglia from the control, NBH inoculated WT and *Apoe*$^{-/-}$ mice, TREM2 level is significantly upregulated in 22L infected mice. There is significantly higher TREM2 expression in 22L WT mice compared to 22L *Apoe*$^{-/-}$ mice despite inverse degree of microglia activation revealed by anti-Iba1 immunostaining. Scale bar: 40 μm.
